# The impact of HIV on cervical cancer elimination in KwaZulu-Natal: a comparative modeling analysis

**DOI:** 10.1093/jnci/djaf364

**Published:** 2025-12-18

**Authors:** Christine L Hathaway, Michaela Hall, Daniël de Bondt, Cara J Broshkevitch, Darcy W Rao, Sydney Klein, Dorothy C Nyemba, Danielle Travill, Sinead Delany-Moretlwe, Karen Canfell, Jan Hontelez, Ruanne V Barnabas

**Affiliations:** Division of Infectious Diseases, Massachusetts General Hospital, Boston, MA, United States; University of Washington School of Medicine, Seattle, WA, United States; Cancer Elimination Collaboration, Sydney School of Public Health, The University of Sydney, NSW, Australia; Department of Public Health, Erasmus MC, Erasmus University Medical Center Rotterdam, Rotterdam, the Netherlands; Department of Epidemiology, University of North Carolina-Chapel Hill, Chapel Hill, NC, United States; Bill and Melinda Gates Foundation, Seattle, WA, United States; Division of Infectious Diseases, Massachusetts General Hospital, Boston, MA, United States; Wits Reproductive Health and HIV Institute, University of the Witwatersrand, Johannesburg, South Africa; Wits Reproductive Health and HIV Institute, University of the Witwatersrand, Johannesburg, South Africa; Wits Reproductive Health and HIV Institute, University of the Witwatersrand, Johannesburg, South Africa; Cancer Elimination Collaboration, Sydney School of Public Health, The University of Sydney, NSW, Australia; Department of Public Health, Erasmus MC, Erasmus University Medical Center Rotterdam, Rotterdam, the Netherlands; Heidelberg Institute of Global Health (HIGH), Heidelberg University Medical Center, Heidelberg, Germany; Division of Infectious Diseases, Massachusetts General Hospital, Boston, MA, United States; Harvard Medical School, Boston, MA, United States

## Abstract

**Background:**

Achieving cervical cancer (CC) elimination requires addressing disparities in CC burden for women living with HIV (WLHIV) and how disparities evolve in the context of antiretroviral therapy (ART) scale-up. To inform CC elimination for high HIV prevalence regions, we modeled the impact of HIV, HIV interventions, and CC interventions in KwaZulu-Natal, South Africa.

**Methods:**

We used 2 independently developed, dynamic compartmental transmission models of HIV and human papillomavirus (*DRIVE* and *Policy1-Cervix-HIV*) calibrated to KwaZulu-Natal. We simulated: a counterfactual without HIV but with observed CC screening and vaccination; and scenarios sequentially adding condom use and voluntary medical male circumcision (VMMC); HIV; observed HIV and CC interventions (status quo); achieving United Nations Programme on HIV/AIDS HIV treatment targets; and achieving World Health Organization (WHO) CC elimination targets. The impact of each scenario was measured as the difference in CC incidence from the previous scenario. Results were reported from 2024 to 2124 as a range between the 2 models; CC elimination was WHO-defined as incidence <4/100 000 women-years.

**Results:**

For the status quo, CC incidence ranged from 61.30 to 78.96/100 000 women-years in 2024, with the highest incidence among WLHIV (126.8-192.0/100 000). HIV contributed an estimated 29.08-48.87 additional cases per 100 000. Neither model predicted elimination under status quo interventions, but achieving HIV treatment and CC elimination targets could reduce incidence to 1.42-6.25/100 000 women-years in 2124.

**Conclusions:**

HIV is associated with a population-level increase in CC incidence. However, scaling up ART coverage and CC interventions is expected to significantly reduce the burden of CC overall and among WLHIV. These conclusions are consistent between both models and strengthened by the comparative modeling approach.

## Introduction

Cervical cancer (CC) remains a leading cause of cancer death among women, with over 660 000 new cases and 348 000 deaths in 2022.^1^ Women living with HIV (WLHIV) have a higher risk, with a 6-fold greater likelihood of CC due to increased susceptibility to human papillomavirus (HPV) and reduced immune clearance.^2-4^ This is observed in KwaZulu-Natal, South Africa, where HIV prevalence among women reached 30.5% in 2018 and over 60% of CC cases affected WLHIV.^3^^,^^5^

To achieve the World Health Organization’s (WHO) 2030 CC elimination target, defined as CC incidence less than 4/100 000 women-years, the strategy includes 90% HPV vaccination coverage among girls by age 15, 70% of women screened by age 35 and 45, and 90% of cases treated.^6^ The WHO and South African screening guidelines recommend more intensive screening for WLHIV every 3-5 years from age 25.^6-9^

HIV control also indirectly impacts CC burden. To end the AIDS epidemic, the Joint United Nations Programme on HIV/AIDS (UNAIDS) launched the 95-95-95 targets: 95% of people living with HIV know their status, 95% of those who know their status are on antiretroviral therapy (ART), and 95% of those treated achieve viral suppression.^10^ Studies have shown that WLHIV on ART have an 18% lower risk of high-risk HPV infection compared to WLHIV who are ART naïve.^11^ Interventions such as voluntary medical male circumcision (VMMC) reduce HIV risk among men by 60%, thereby reducing CC risk among women by 58%.^12-14^ Similarly, condom use has been associated with reduced HIV and HPV acquisition.^15^

Mathematical modeling has informed global HIV programs^16^^,^^17^ and the WHO strategies for CC elimination.^18^^,^^19^ However, due to the interactions between HIV and HPV, the combined impact of these programs on CC remains unclear. A comparative modeling approach, in which multiple models are developed independently and run on the same scenarios, provides a range of potential results and is informative for policy.^20^ This analysis uses comparative modeling to (1) estimate the effect of past HIV interventions on CC burden, and (2) assess the projected impact of scaling up ART, HPV vaccination, and screening in KwaZulu-Natal, South Africa.

## Methods

### Description of models

This study uses comparative modeling, where models were developed, parameterized, and calibrated independently. These models generated outcomes for the same set of scenarios, and the results were compared.

Two mathematical models were used: *DRIVE* and *Policy1-Cervix-HIV* are dynamic, deterministic HIV and HPV transmission compartment models. Both were calibrated to KwaZulu-Natal, South Africa, have been used to inform the 2021 WHO screening and treatment guidelines for WLHIV, and have been described in published papers.^8^^,^^21-25^ Model description, calibration, validation, and sensitivity analyses are detailed in the Supplementary Material (Figures S1-S4).

Both models represent HIV and HPV interactions, whereby HIV infection increases the risk of HPV acquisition and the rate of disease progression, and are calibrated to reproduce observed population-level HPV and HIV dynamics. Notable differences between the models are that *DRIVE* simulates 2 HPV groups (16/18/31/33/45/52/58 and other high-risk types), while *Policy1-Cervix-HIV* simulates 3 HPV groups (16/18, 31/33/45/52/58, and other high-risk types). Additionally, *DRIVE* models HIV viral load as unsuppressed or fully suppressed, while *Policy1-Cervix-HIV* includes partial and full HIV viral suppression with ART use. Comparisons of both models are summarized in Table S1.

### Scenarios

To assess the effect of historical HIV and HIV-related interventions on the current CC burden, scenarios began with a counterfactual baseline of no HIV, no HIV-related interventions (no condoms, no ART, and VMMC maintained at low 1960 levels), and as-observed CC interventions. Each subsequent scenario added non-ART HIV-related interventions (condoms and VMMC as historically observed), HIV beginning in 1980, and ART coverage as-observed beginning in 2004 (status quo).

To assess the effect of ART, HPV vaccination, and CC screening scale-up, we ran the status quo up to 2024, and layered an additional intervention for each subsequent scenario to ultimately reach the UNAIDS and WHO targets: ART scale-up to 95-95-95 HIV treatment targets by 2030; HPV vaccination coverage scale-up to 90% coverage in 2024; screening with HPV-DNA at a 70% coverage in 2024 with single-visit screen-and-treat with thermal ablation; and a switch to repeat HPV-DNA testing (twice per lifetime for women without HIV and every 5 years for WLHIV).^6^^,^^10^  Table 1 lists the scenarios.

**Table 1.** HIV and HPV-related intervention modeled scenarios.

Scenario	Description
*The effect of HIV and HIV-related interventions on the current burden of cervical cancer*	
No HIV, no HIV-related interventions (S0)	No HIV, VMMC set to low 1960 values with no scale-up,^a^ 0% condom coverage,^b^ observed HPV vaccination,^c^ and observed cervical cancer screening with cytology^d^
+ non-ART HIV-related interventions (S1)	S0 + VMMC scale-up as observed,^a^ condom coverage scale-up as observed^a^
+ HIV (S2)	S1 + HIV introduced in the models
+ observed ART (S3 - *status quo*)	S2 + ART coverage as observed (viral suppression for people living with HIV is 67% for females and 60% for males)^e^
*The effect of ART, HPV vaccination, and cervical cancer screening scale-up on projected cervical cancer incidence*	
+ 95-95-95 ART scale-up (S4)	S3 + ART coverage scaled up to 95-95-95 targets linearly from 2024 to 2030 (viral suppression for people living with HIV is 85.7% for both males and females)
+ HPV vaccination scale-up (S5)	S4 + HPV vaccination coverage scale-up to 90% in 2024
+ HPV DNA and screening coverage scale-up (S6)	S5 + switch to HPV DNA screening in 2024, screening coverage scaled up linearly to 70% in 2030, single visit screen and treat^f^
+ repeat screening (S7)	S6 + twice per lifetime screen for women without HIV at age 35-39 and 45-49, screening every 5 years for WLHIV starting from age 25^g^

a See Table S1 for more detail on coverage levels.

b Condom use is assumed to be in response to the HIV epidemic.

c HPV vaccination is introduced in 2014 with the bivalent vaccine at a coverage of 57%. Assume a switch to the nonavalent vaccine in 2024.

d Cervical cancer screening with cytology is introduced in 2000 with once in a lifetime screening at age 35-39. Screening coverage is scaled up from 0% to 18% from 2000-2003, then 18% to 48% from 2003-2016, then held at 48% for the rest of the time horizon. Assume treatment with cryotherapy/LLETZ (28% loss-to-follow-up for colposcopy, 49% loss-to-follow-up for treatment with anyone with confirmed CIN).

e ART is introduced in 2004. From 2004 to 2017, viral suppression is based on observed data. ART coverage is scaled up linearly from observed levels in 2017 to the 90-90-90 targets in 2030, but held at 2024 levels from 2024 to 2124. This equates to a viral suppression coverage of 67% for females and 60% for males.

f 5% loss-to-follow-up for thermal ablation, 20% loss-to-follow-up for LLETZ.

g The WHO recommends screening every 3 years for WLHIV, but because of the *DRIVE* and *Policy1-Cervix-HIV* models’ 5-year age groups, screening is applied every 5 years for WLHIV.

In these scenarios, we defined ART coverage as the percentage of all persons living with HIV above age 10 who are virally suppressed. Both models introduced ART in 2004, and viral suppression was based on observed data up to 2017.^26-30^ From 2017 to 2024, ART coverage was scaled up linearly from observed levels in 2017 to the UNAIDS 90-90-90 treatment targets in 2030 but then held at intermediate 2024 levels, equating to a percentage of viral suppression of 67% for females and 60% for males.^10^ The ART scale-up scenario utilized this same linear scale-up from 2017-2030, but reached the UNAIDS 95-95-95 targets in 2030, equating to a percentage of viral suppression of 85.7% for both females and males.^10^

All scenarios also introduced the bivalent HPV vaccine (HPV16/18) in 2014 with 57% coverage, consistent with last-dose coverage levels of the school-based national program in South Africa.^31^ We made an optimistic assumption of a transition to the nonavalent vaccine (HPV16/18/31/33/45/52/58) in 2024 to evaluate the maximum effect of the HPV vaccine. Vaccine efficacy was 100% for all individuals regardless of HIV status, as assumed in prior modeling studies. Status quo screening began in 2000 with cytology and one lifetime screen at age 35-39, consistent with previous studies.^22^^,^^24^ Screening coverage was scaled up from 0% to 18% from 2000 to 2003, then 18%-48% from 2003 to 2016, then held at 48%.^32^ Treatment for cervical precancer was cryotherapy/LLETZ.

### Outcomes

Both models reported results for a 100-year time horizon (2024-2124). The main outcome was age-standardized CC incidence, standardized using the 2015 World Female Population, and stratified for all women, WLHIV, and women without HIV.^33^

To assess the contribution of HIV and HIV-related interventions on the current burden of CC, we used Scenario 0 (no HIV, no HIV-related interventions) to Scenario 3 (status quo) and measured the difference in CC incidence in 2024 between each scenario and its adjacent previous scenario.

To assess the contribution of ART, HPV vaccination, and CC screening scale-up on projected CC incidence, we used Scenario 3 (status quo) to Scenario 7 (meeting 95-95-95 UNAIDS HIV treatment and WHO CC elimination recommendations) and measured the difference in CC incidence in 25-year intervals from 2024 to 2124 between each scenario and its adjacent previous scenario. To compare macro trends in model dynamics after intervention scale-up, for each time point, we averaged the incidence over the next 10 years and defined that as the estimated incidence for that timepoint.

Results were described as a range of the median results of the 2 models. The *DRIVE* model reported uncertainty as a range of the 25 best-fitting parameter sets. The *Policy1-Cervix-HIV* model reported uncertainty in a probabilistic sensitivity analysis of the status quo and full scale-up scenario, the methods of which are described in further detail in the Supplementary Material.

### Elimination thresholds

For the scale-up scenarios, we estimated the year of CC elimination using thresholds previously defined by the WHO as CC incidence below 4/100 000 women-years (henceforth described as the “<4 threshold”) or below 10/100 000 women-years (henceforth described as the “<10 threshold”).^6^ The year of elimination was defined as the first year in which age-standardized CC incidence fell below these thresholds.

## Results

### Current and projected burden of cervical cancer with observed interventions

**Figure 1.**
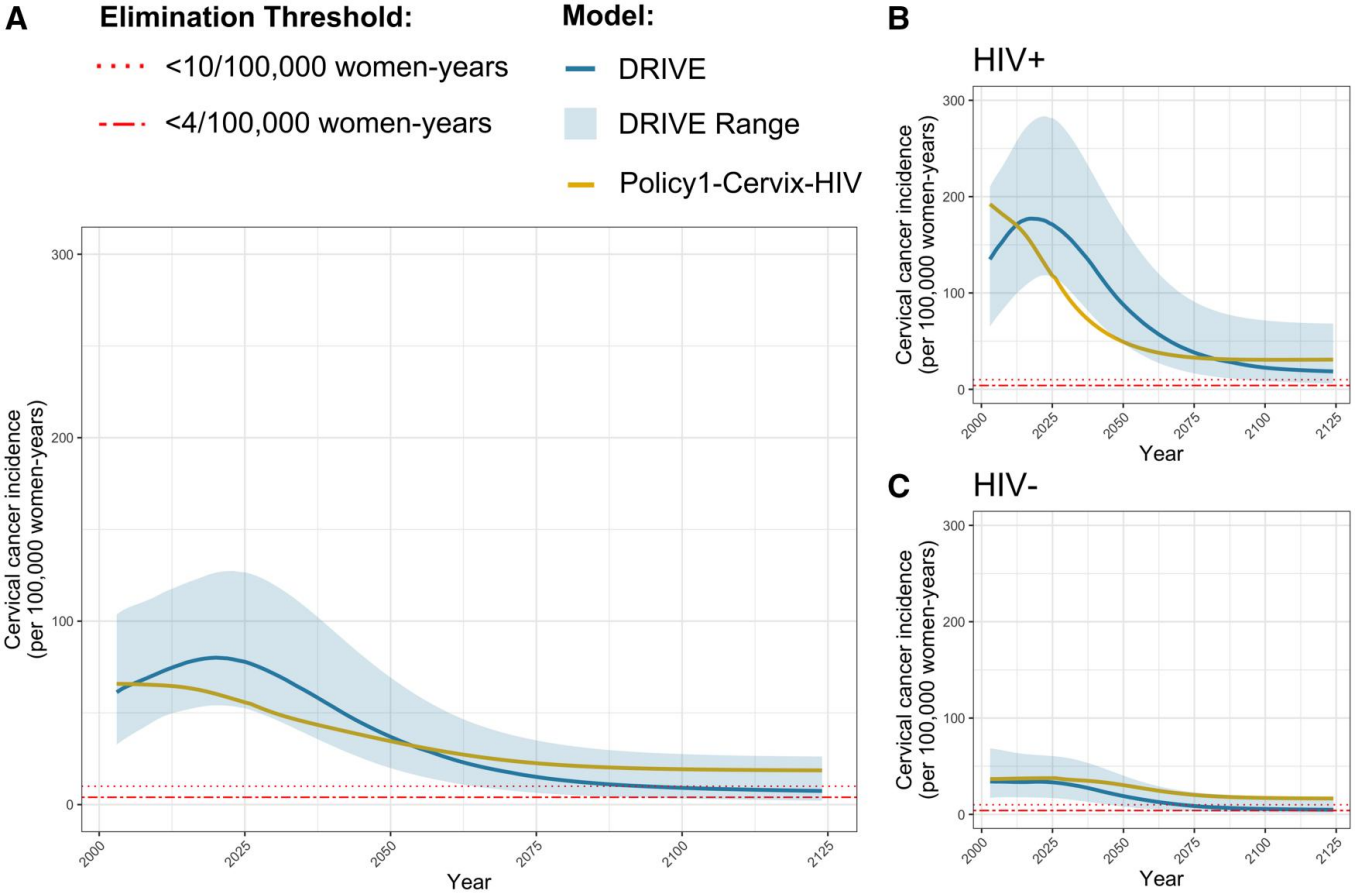
Age-standardized cervical cancer incidence for the status quo scenario of observed ART coverage, HPV vaccination, and cervical cancer screening maintained for the full time horizon to 2124, for **(A)** all women, **(B)** WLHIV, and **(C)** women without HIV. The dotted lines represent the cervical cancer elimination thresholds of 10 and 4 per 100 000 women-years. Both models report median results, and the *DRIVE* model also reports uncertainty as a range of results from 25 of the best-fitting parameter sets.

With observed ART coverage of 67% for females and 60% for males, HPV vaccination coverage of 57%, and once-per-lifetime CC screening with cytology at a 48% coverage, the models predicted a median age-standardized CC incidence in 2024 of 61.30 (*Policy1-Cervix-HIV)* to 78.96 (Range: 53.06-126.84) (*DRIVE)* per 100 000 women-years (Figure 1). The CC burden was higher for WLHIV, with an incidence of 122.38-172.33/100 000 women-years, more than 3-5 times higher than the incidence for women without HIV of 33.42-37.57/100 000 women-years.

Maintaining these interventions for 100 years, neither model predicted CC elimination below the <4 threshold for WLHIV or women without HIV. The incidence in 2124 was 7.46-18.65/100 000 women-years for all women, 18.60-30.85/100 000 women-years for WLHIV, and 4.87-16.56/100 000 women-years for women without HIV.

### Contribution of HIV and HIV-related interventions to current rates

**Figure 2.**
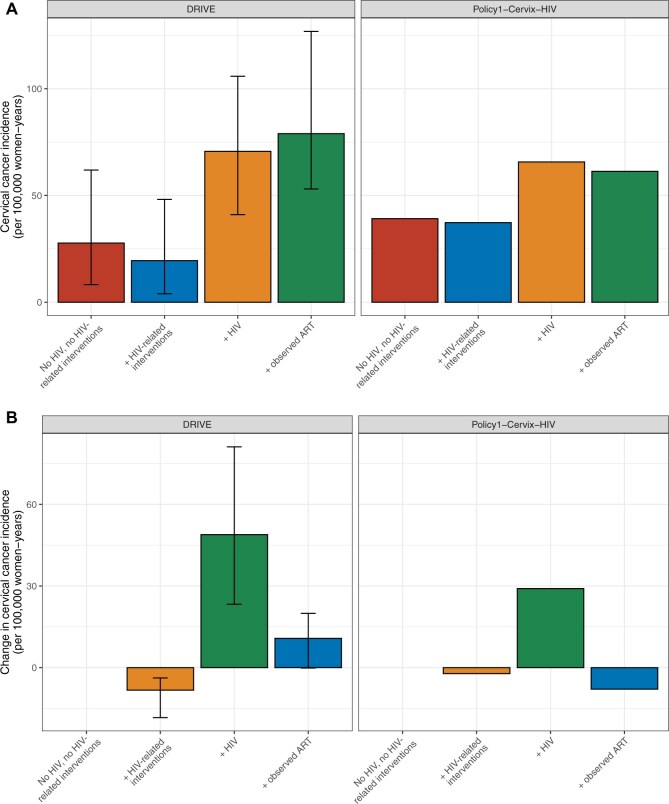
**A)** Age-standardized cervical cancer incidence in 2024 for scenarios evaluating the impact of HIV-related interventions and ART on the current burden of cervical cancer in KwaZulu-Natal. **B)** The difference in age-standardized cervical cancer incidence compared to the previous scenario in 2024. Both models report median results and the error bars for the *DRIVE* model indicate the range of results from 25 of the best-fitting parameter sets.

With observed CC interventions and a counterfactual scenario of no HIV and no HIV-related interventions, the models predicted that the incidence of CC in 2024 would be 27.71-39.16/100 000 women-years (Figure 2 and Table 2). Both models predicted that the introduction of condoms and expansion of VMMC contributed to absolute population-level reductions in CC incidence of 2.19-8.26/100 000 women-years in 2024.

**Table 2.** The impact of HIV, ART, HPV vaccination, and cervical cancer screening scale-up on the current and predicted burden of cervical cancer in KwaZulu-Natal from 2024 to 2124.

		DRIVE				Policy1-Cervix-HIV			
Scenario	Year	Age-standardized cervical cancer incidence (per 100 000 women-years); Median (range)^a^	Percent reduction in incidence compared to 2024 (%); Median (range)^b^	Contribution of intervention to cervical cancer incidence (per 100 000 women-years); Median (range)^c^	Elimination year (<10 threshold, <4 threshold); Median^d^	Age-standardized cervical cancer incidence (per 100 000 women-years); Median^a^	Percent reduction in incidence compared to 2024 (%); Median^b^	Contribution of intervention to cervical cancer incidence (per 100 000 women-years); Median^c^	Elimination year (<10 threshold, <4 threshold); Median^d^
*The effect of HIV and HIV-related interventions on the current burden of cervical cancer*									
No HIV (S0)	2024	27.71 (8.21, 61.89)	–	–	–	39.16	–	–	–
+ non-ART HIV-related interventions (S1)	2024	19.47 (3.98, 48.16)	–	−8.26 (−18.36, −3.79)	–	37.25	–	−2.19	–
+ HIV (S2)	2024	70.68 (41.03, 105.83)	–	48.87 (23.31, 81.16)	–	65.71	–	29.08	–
+ observed ART (S3-status quo)	2024	78.96 (53.06, 126.84)	–	10.78 (−0.16, 19.94)	–	61.30	–	−7.90	–
*The effect of ART, HPV vaccination, and cervical cancer screening scale-up on projected cervical cancer incidence*									
+ observed ART (S3-status quo)	2024	73.13 (53.06, 126.84)	0.00 (0.00, 0.00)	–	2099, -	51.83	0.00	–	-, -
	2049	32.61 (20.90, 71.60)	51.02 (36.91, 68.05)	–		32.34	37.84	–	
	2074	13.73 (6.68, 35.63)	82.21 (66.66, 90.37)	–		21.76	59.75	–	
	2099	8.80 (3.35, 27.62)	89.09 (72.44, 95.05)	–		19.12	65.93	–	
	2124	7.46 (2.15, 26.2)	90.89 (73.71, 97.11)	–		18.65	67.10	–	
+ 95-95-95 ART scale-up (S4)	2024	73.80 (53.07, 126.88)	0.00 (0.00, 0.00)	0.03 (0.01, 0.04)	2074, 2124	51.52	0.00	−0.32	-, -
	2049	31.68 (19.72, 72.44)	52.79 (36.57, 70.32)	−2.36 (−5.63, 0.15)		30.42	41.05	−1.92	
	2074	9.35 (3.63, 28.25)	87.69 (72.25, 94.94)	−8.46 (−14.73, −4.77)		19.08	64.17	−2.68	
	2099	4.47 (1.06, 18.74)	94.16 (79.54, 98.48)	−8.63 (−17.01, −3.60)		15.75	71.69	−3.37	
	2124	3.50 (0.32, 16.65)	95.47 (81.45, 99.59)	−7.73 (−17.74, −2.30)		14.94	73.62	−3.71	
+ HPV vaccination scale-up (S5)	2024	73.71 (53.07, 126.88)	0.00 (0.00, 0.00)	0.00 (0.00, 0.00)	2074, 2099	51.52	0.00	0.00	-, -
	2049	27.62 (17.86, 64.99)	56.02 (40.33, 72.79)	−2.96 (−7.00, −1.57)		28.93	42.73	−1.49	
	2074	6.32 (2.89, 17.18)	90.78 (83.10, 96.00)	−3.04 (−11.03, −0.68)		15.44	70.26	−3.64	
	2099	3.09 (0.81, 9.81)	95.19 (91.15, 98.95)	−1.00 (−8.82, −0.10)		11.89	78.54	−3.86	
	2124	2.78 (0.21, 8.82)	95.89 (92.14, 99.73)	−0.25 (−7.74, −0.01)		11.19	80.25	−3.75	
+ HPV DNA and screening coverage scale-up (S6)	2024	70.81 (52.98, 126.59)	0.00 (0.00, 0.00)	−0.09 (−0.17, −0.06)	2074, 2099	42.67	0.00	−8.85	2099, -
	2049	21.07 (13.95, 49.69)	62.63 (47.55, 76.63)	−4.36 (−8.69, −2.13)		19.34	62.04	−9.59	
	2074	4.38 (1.95, 12.13)	92.68 (86.45, 96.75)	−1.18 (−2.53, −0.48)		10.65	79.42	−4.79	
	2099	2.45 (0.51, 7.58)	95.84 (92.38, 99.20)	−0.44 (−1.19, −0.15)		8.04	85.47	−3.85	
	2124	2.28 (0.12, 6.91)	96.56 (93.18, 99.80)	−0.30 (−1.02, −0.05)		7.57	86.65	−3.62	
+ repeat screening (S7)	2024	55.36 (49.48, 117.25)	0.00 (0.00, 0.00)	−5.54 (−9.35, −3.47)	2049, 2074	36.35	0.00	−6.31	2074, -
	2049	9.33 (5.92, 25.16)	83.79 (77.78, 91.39)	−14.11 (−22.94, −7.12)		15.35	69.06	−4.00	
	2074	2.26 (0.79, 7.06)	96.86 (93.19, 98.75)	−2.47 (−4.76, −0.98)		8.69	82.53	−1.95	
	2099	1.53 (0.15, 5.54)	97.96 (94.37, 99.80)	−0.84 (−1.95, −0.26)		6.58	87.65	−1.46	
	2124	1.42 (0.04, 5.27)	98.19 (94.63, 99.96)	−0.54 (−1.60, −0.07)		6.25	88.53	−1.32	

a For Objective 2 (the effect of ART, HPV vaccination, and cervical cancer screening scale-up on projected cervical cancer incidence), cervical cancer incidence was averaged over each time point and the subsequent 10 years.

b Calculated only for Objective 2 (the effect of ART, HPV vaccination, and cervical cancer screening scale-up on projected cervical cancer incidence). Dashes indicate that the value was not calculated for those scenarios.

c Dashes are shown for the first scenarios in the objective since there is no previous scenario to compare to.

d Dashes signify that the elimination threshold was not reached within 100 years.

HIV had the greatest impact on the current burden of CC, with increases in incidence of 29.08-48.87/100 000 women-years compared to the previous scenario. Without the introduction of ART, the current CC incidence in the population was predicted to be 65.71-70.68/100 000 women-years.

The models differed in their estimated impact of ART on the current CC burden. *DRIVE* predicted that ART increased the current CC burden (by 10.78/100 000 women-years), while *Policy1-Cervix-HIV* predicted that ART reduced the current CC burden (by 7.90/100 000 women-years) compared to the previous scenario.

### Contribution of ART, HPV vaccination, and cervical cancer screening scale-up to future rates

**Figure 3.**
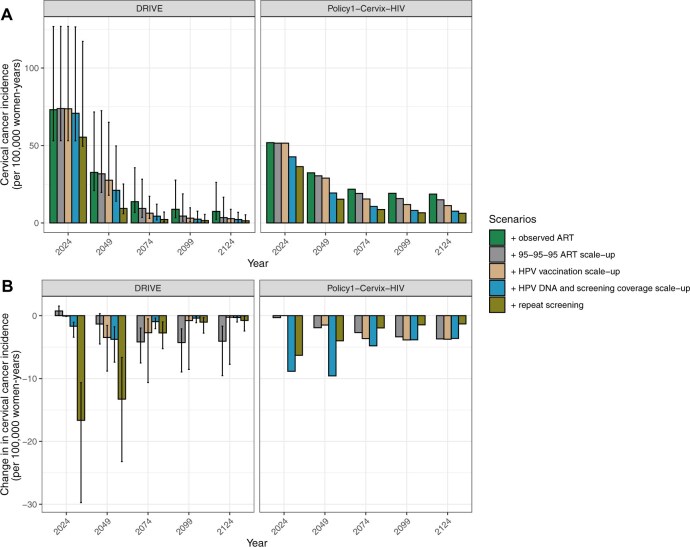
**A)** Age-standardized cervical cancer incidence from 2024 to 2124 in 25-year intervals for scenarios evaluating the impact of ART, HPV vaccination, and cervical cancer screening scale-up on the future burden of cervical cancer in KwaZulu-Natal. **B)** The difference in age-standardized cervical cancer incidence compared to the previous scenario for each time point. Both models report median results and the error bars for the DRIVE model indicate the range of results from 25 of the best-fitting parameter sets.

Despite differences in the estimated impact of ART, both models were aligned in estimates of the long-term CC burden. The models estimated that current ART coverage levels reduced CC incidence by 67.10%-90.89% over 100 years (incidence of 7.46-18.65/100 000 women-years in 2124), shown in Figure 3 and Table 2. Scaling up ART to reach the UNAIDS treatment targets reduced CC incidence to 3.50-14.94/100 000 women-years in 2124. ART scale-up had a moderate impact on CC incidence over the first 25 years, contributing to CC incidence reductions by 1.92-2.36/100 000 women-years by 2049. However, the impact was more pronounced in the long-term, with reductions of 2.68-8.46/100 000 women-years in 2074, and 3.71-7.73/100 000 women-years in 2124.

HPV vaccination coverage scale-up was predicted to accelerate reductions in CC incidence, contributing to reductions by 1.49-2.96/100 000 women-years in 2049, and 0.25-3.75/100 000 women-years in 2124 (compared to 95-95-95 ART scale-up). The addition of HPV vaccination resulted in CC incidence of 2.78-11.19/100 000 women-years in 2124. Scaling up CC screening to 70% coverage using HPV-DNA and thermal ablation treatment further reduced CC incidence, contributing to an incidence reduction by 4.36-9.59/100 000 women-years in 2049, and 0.30-3.62/100 000 women-years in 2124 (compared to HPV vaccination scale-up).

A final increase in screening frequency to twice-lifetime for women without HIV and every 5 years for WLHIV had the greatest short-term reductions in CC incidence by 5.54-6.31/100 000 women-years in 2024, and 4.00-14.11/100 000 women-years in 2049 (compared to the previous scenario). Overall, if KwaZulu-Natal can achieve the 95-95-95 UNAIDS targets for HIV treatment and 90-70-90 WHO targets for CC elimination, CC incidence was predicted to reach 1.42-6.25/100 000 women-years in 2124, a reduction of 88.53%-98.19% over 100 years.

### Impact of interventions on cervical cancer elimination

With the status quo, *DRIVE* predicted CC elimination (<10 threshold) in 2099, while neither model predicted incidence to fall below the <4 elimination threshold (Table 2). However, *DRIVE* predicted that meeting 95-95-95 UNAIDS targets would accelerate incidence reduction to the <10 threshold in 2074, and <4 threshold in 2124. The addition of HPV vaccination did not affect the timing of reaching the <10 threshold, but it accelerated the time to the <4 threshold to 2099. The time to elimination was unchanged with the switch to HPV-DNA/thermal ablation/increased coverage. If KwaZulu-Natal can achieve the 95-95-95 UNAIDS and 90-70-90 WHO targets, CC elimination was estimated to be achieved in 2049 (<10 threshold) and 2074 (<4 threshold).

*Policy1-Cervix-HIV* predicted that the <10 threshold would first be achieved in 2099 with the addition of HPV-DNA/thermal ablation/coverage scale-up. A full scale-up to UNAIDS HIV and WHO CC targets was expected to reach the <10 threshold in 2074, while the <4 threshold was not reached within 100 years.

**Figure 4.**
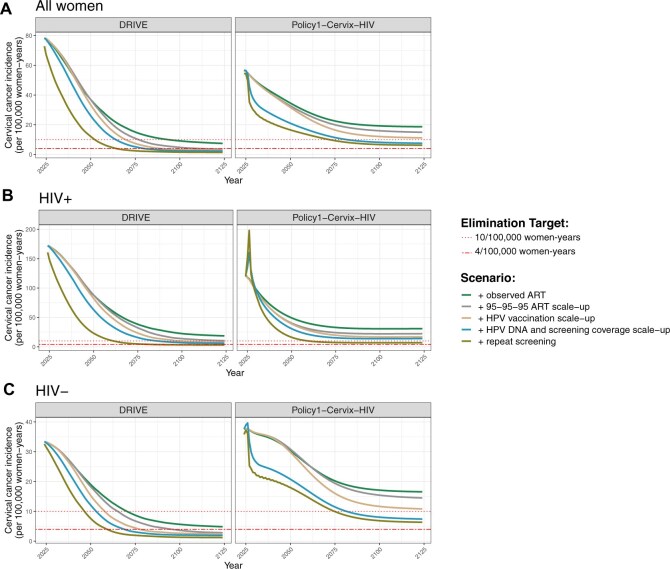
Age-standardized cervical cancer incidence from 2024 to 2124 for the ART, HPV vaccination, and cervical cancer screening scale-up scenarios for **(A)** all women, **(B)** WLHIV, and **(C)** women without HIV. The dotted lines represent the elimination thresholds of 10 and 4 per 100 000 women-years.

Neither model predicted CC elimination for WLHIV under the status quo. However, with full ART and CC intervention scale-up (S7), incidence dropped below 10/100 000 women-years in 2062 (*DRIVE*) and 2060 (*Policy1-Cervix-HIV*), while the <4 threshold was reached in 2083 for *DRIVE* (Figure 4). Cervical cancer elimination was reached sooner for women without HIV in *DRIVE*, crossing the <10 threshold in 2047 and the <4 threshold in 2060. *Policy1-Cervix-HIV* crossed the <10 threshold in 2075 and did not cross the <4 threshold for both women with and without HIV within 100 years. Supplemental results for both models are presented in Figures S5-S14.

## Discussion

This study used comparative modeling to assess the historical impact of HIV and the projected effects of scaling up ART, HPV vaccination, and screening on CC burden in KwaZulu-Natal, South Africa, a region with high HIV prevalence. Comparative modeling helps identify how model design and assumptions affect outcomes, aiding public health decision-making. While focused on KwaZulu-Natal, findings are broadly relevant to other high HIV prevalence regions.

Historically, HIV had the greatest impact, increasing CC incidence by 29.08-48.87/10 000 women-years in 2024 compared to no HIV. The impact of ART varied. In *DRIVE*, it led to a temporary increase in incidence (+10.78, range: −0.16 to 19.94) as women lived longer with HIV, aligning with observations from previous modeling studies (Figures S13 and S14).^25^^,^^34^ In *Policy1-Cervix-HIV*, ART led to a decrease of 7.90/100 000 women-years, reflecting faster reductions in HIV prevalence (Figure S9). These differences stem from model assumptions: *DRIVE* assumes perfect ART adherence, leading to greater survival among WLHIV, while *Policy1-Cervix-HIV* models incomplete ART adherence, resulting in slower improvements in survival and therefore a diminished temporary spike in incidence.

Despite these differences, both models are aligned in the observed longer-term impact of ART. Scaling up ART to the 95-95-95 UNAIDS targets reduced CC incidence by 3.71-7.73/100 000 women-years in 2124, an additional 4.58%-6.52% reduction compared to the status quo. Both models showed short-term gains from screening scale-up, medium-term gains from HPV vaccination, and long-term gains from ART (Figure 3B). These trends align with prior modeling in South Africa, where initial benefits from screening diminished as vaccination-induced herd immunity reduced HPV prevalence.^35^

The models ultimately estimated that meeting UNAIDS and WHO targets could reduce CC incidence to 1.42-6.25/100 000 women-years in 2124. This is consistent with previous modeling studies: van Schalkwyk et al. estimated an incidence of 4.0 (2.3-6.2)/100 000 women in 2120, and Brisson et al. estimated an equilibrium incidence of 1.4/100 000 women in 2100-2120.^18^^,^^35^

*Policy1-Cervix-HIV* is more conservative in its predictions for CC elimination, with none of the scenarios reaching the <4 threshold, and only the screening coverage scale-up and repeat screening scenarios crossing the <10 threshold. This may be due to higher starting HPV prevalence and CC incidence, different assumptions related to HPV natural immunity, ART effects on HPV progression, and a higher proportion of prevalent oncogenic HPV not being targeted by the HPV vaccine, as the 2 models were calibrated to different datasets for underlying HPV type distribution. This last factor was assessed in a partial rank correlation analysis and found to be highly influential on steady state predictions of CC incidence rates, given assumed high coverage of HPV vaccination. In multivariate sensitivity analysis, parameter sets where the distribution of prevalent HPV types skewed towards vaccine-preventable genotypes were more likely to reach CC elimination (Table S2, Figures S15 and S16). In multivariate sensitivity analysis, steady state CC incidence rates for scenarios S3 and S4 ranged from 3.4 to 8.0 and 3.0 to 7.2, respectively.

A key strength of this study is the comparative modeling approach. Despite differences between the 2 models’ structure and parameters, they showed consistent trends in CC incidence for HIV and CC-related interventions. Additionally, both models dynamically accounted for HIV and HPV coinfection, allowing us to simulate targeted interventions for WLHIV and stratify results by HIV status. These strengths lend themselves to future estimates that have a greater degree of confidence than if results were reported for a single model.

This analysis also had several limitations. Both models were calibrated to 2018 GLOBOCAN estimates due to the lack of CC incidence data in KwaZulu-Natal.^36^ Any biases in the GLOBOCAN estimates would therefore be propagated in the models. We also did not account for pre-exposure prophylaxis (PrEP), with 2020 coverage in South Africa reported at 17/100 000 people.^37^ Without modeling PrEP, we likely overestimated CC incidence. We also assumed ambitious scale-up scenarios that happen rapidly in 2024, so predictions for CC elimination are likely optimistic. Finally, we did not account for South Africa switching to single-dose HPV vaccination. In both models, we assumed 100% vaccine efficacy with 2 doses, but it is unlikely that single-dose efficacy will affect the results due to the comparable single-dose efficacy found in clinical trials.^38^^,^^39^ Future studies accounting for varying HPV vaccine efficacy by HIV status are warranted, especially for single-dose vaccination. A single-dose strategy will likely have significant cost implications, so analyses exploring the cost-effectiveness of ART, HIV prevention, HPV vaccination, and CC screening scale-up are also warranted.

In conclusion, while the HIV epidemic has significantly increased the CC burden in KwaZulu-Natal, scaling up ART, HPV vaccination, and CC screening is predicted to reduce CC incidence and make elimination possible. However, realizing this goal will require a more integrated approach that aligns HIV prevention and treatment with CC control strategies. Strengthening collaboration between HIV and CC programs is essential to optimize programmatic efficiency and improve health outcomes for WLHIV. Additionally, further research is warranted to identify and address implementation challenges to CC screening and the treatment of pre-invasive disease in WLHIV.

## Supplementary Material

djaf364_Supplementary_Data

## Data Availability

Model inputs and methods are presented in the Supplementary Material. Model code is available upon reasonable request with submission of a concept note to the authors. The model output data used for this analysis are available in a GitHub repository: https://github.com/clhchristine/Impact_of_HIV_ART_CC_Comparative_Modeling
