# Supplementary material for: The impact of HIV on cervical cancer elimination in KwaZulu-Natal: a comparative modeling analysis
Source: J Natl Cancer Inst. 2025 Dec 18;118(3):531–40. doi: 10.1093/jnci/djaf364 (PMC13017777; doi:10.1093/jnci/djaf364)
Supplement: djaf364_Supplementary_Data [file djaf364_supplementary_data.zip › HIVHPV Appendix_Final.pdf]

Supplementary Material

Table of Contents

I. Overview of models 2

I.a. DRIVE 2

I.b. Policy1-Cervix-HIV 3

I.c. Summary of key assumptions between models 5

II. Model outcomes 7

II.a. Model fit to observed data 7

II.b. Model outcome comparisons 8

II.c. Sensitivity analyses 15

II.c.i. Policy1-Cervix-HIV Multivariate Sensitivity Analysis 15

III. Reporting 17

III.a. HPV-FRAME checklist: DRIVE Model 17

III.b. HPV-FRAME checklist: Policy1-Cervix-HIV Model 21

VI. References 24

I. Overview of models

Two deterministic, transmission dynamic, compartmental models *DRIVE* (developed by Massachusetts General Hospital) and *Policy1-Cervix-HIV* (developed by the Daffodil Centre), both calibrated to KwaZulu-Natal (KZN), South Africa, were used to assess the population-level impact of HIV, ART, and cervical cancer interventions on the cervical cancer burden in KwaZulu-Natal, a region with a high prevalence of HIV. Each model is structured to reflect demography, sexual behavior, HIV and HPV infection, HIV and HPV natural history, interactions between simultaneous HIV and HPV infections, and interventions to prevent and treat HIV and HPV/cervical cancer. The summary of each model is as follows.

I.a. DRIVE

DRIVE is a model for the province of KwaZuluNatal. Briefly, *DRIVE* is a deterministic transmission dynamic model that simulates HIV and HPV transmission (2 groups of high-risk genotypes: HPV16/18/31/33/45/52/58, non-vaccine high-risk HPV types), HIV disease progression and HPV-induced cervical carcinogenesis associated with each type group in a heterosexual population that is stratified by sex, 5-year age groups, and sexual activity. Each health state aims to represent the underlying true health state of the simulated individuals (as opposed to a diagnosed state) such as infection status, grade of cervical intraepithelial neoplasia (CIN1, 2, 3), and cancer stage (see flowchart below). The model takes into account the multiple interactions between HIV and HPV infection and disease progression, namely it is assumed that HIV increases the risk of HPV acquisition and disease progression to cervical cancer. WLHIV on ART and virally suppressed are assumed to experience rates of HPV acquisition and disease progression comparable to HIV negative women, but their HPV clearance rate, disease regression rates, and cervical cancer-associated mortality are only partially restored (and are assumed similar to untreated WLHIV with high (>500) CD4 count). The model also assumes that condom use is effective against HPV acquisition. Male circumcision is assumed to have no effect on HPV (details in Table S1 below).

The model represents variation in the levels of HIV interventions such as condom use, male circumcision and HIV treatment (ART) of people living with HIV (PLHIV) over time since the beginning of the HIV epidemic in 1980. The model assumes that all individuals treated with ART are virally suppressed. The model also represents HPV vaccination (starting in 2020) and baseline cervical cancer screening and treatment (starting in 2000).

The risk of HPV acquisition among susceptible individuals depends on their sexual activity, the prevalence of infection among partners, HIV and ART status, and level of condom use and HPV vaccination interventions. The transition rates between HPV health states depend on sex, age, HPV type group, and HIV/ART status. The risk of HIV acquisition depends on sexual activity, the prevalence of infection and viral load among partners, and level of HIV interventions. The transition rates between HIV health states depend on sex and age.

Additional details of the *DRIVE* model and parameter used can be found in the Supplemental Material of a previous publication.[1]

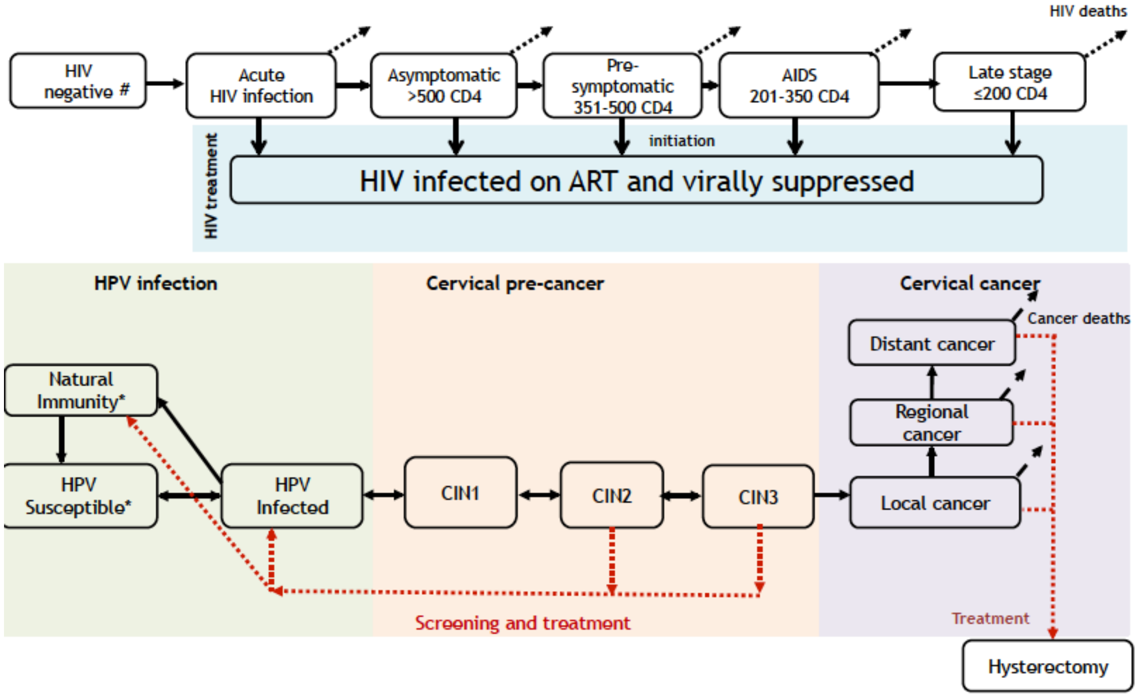

*Figure S1: Flowchart of the model representing the HIV and HPV/CC health states represented in the model. Arrows represent flows between health states. *HPV vaccination is effective only for individuals in these stages. Once received, vaccination status is tracked through subsequent transitions. #HIV susceptible men are stratified by circumcision status, with circumcision decreasing the risk of HIV acquisition.*

I.b. Policy1-Cervix-HIV

The *Policy1-Cervix-HIV* model platform comprises a collection of tools including a HIV and HPV transmission and natural history model, and modules for HIV and cervical cancer control, which has been adapted for a range of different countries and contexts.[2-4] The *Policy1-Cervix-HIV* model is a deterministic transmission-dynamic compartment model of sexual behaviour, HIV and HPV infection and natural history, which captures simultaneous HIV and HPV infections, including for multiple HPV types, and incorporates comprehensive demographic, sexual behaviour and natural history assumptions by 5-year age-groups. It also accounts for voluntary medical male circumcision (VMMC) and anti-retroviral therapy for men and women living with HIV. Figure S2 depicts a diagram of key compartments of the *Policy1-Cervix-HIV* platform with key model assumptions described in further detail in Table S1.

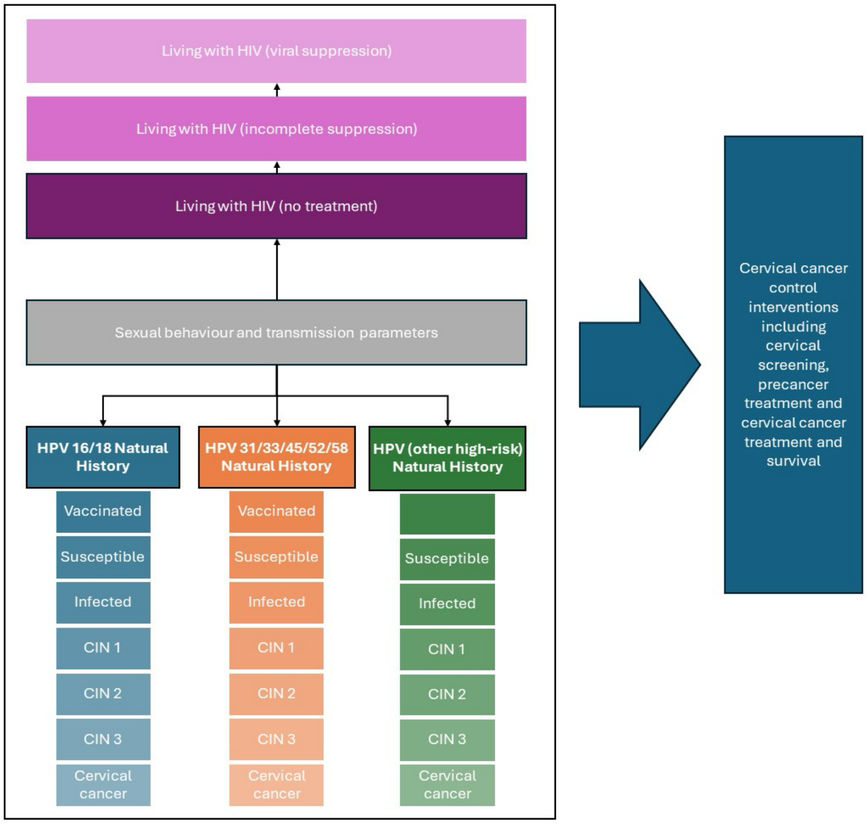

Figure S2: Flowchart of the model representing the HIV and HPV/CC health states represented in Policy1-Cervix-HIV.

Model parameters specific to KwaZulu-Natal include demography information (fertility and mortality rates), obtained from the United Nations Population Division (2022) reports for South Africa and uptake of behavioural interventions as described in Table S1. Model parameters for HPV and HIV natural history, including the interaction between HIV and HPV, are as described in previous publications as they are assumed to remain consistent irrespective of the setting being modelled.[5,6] Age and sex-specific assumptions for sexual behaviour in KwaZulu-Natal (Figure S3) were found via calibration to (1) 2005 age-specific HIV prevalence in Kwa Zulu Natal (Figure S4), and (2) age-specific cervical cancer incidence in KwaZulu-Natal in 2018 (Figure S5). Following calibration, the model was validated against observed age- and HIV-status specific HPV prevalence in South Africa in 2002 (Figure S6).

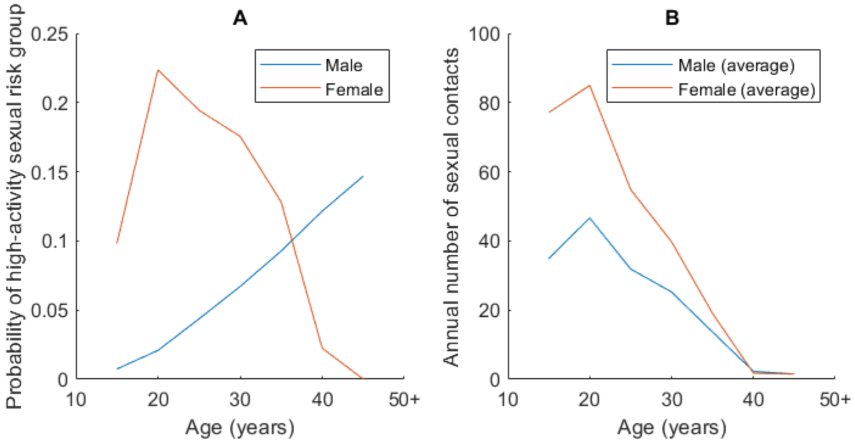

Figure S3: (A) Age-specific probability that a male or female will be in a high-activity risk group for sexual behaviour, and (B) the average number of annual sexual contacts for males and females assumed by Policy1-Cervix-HIV.

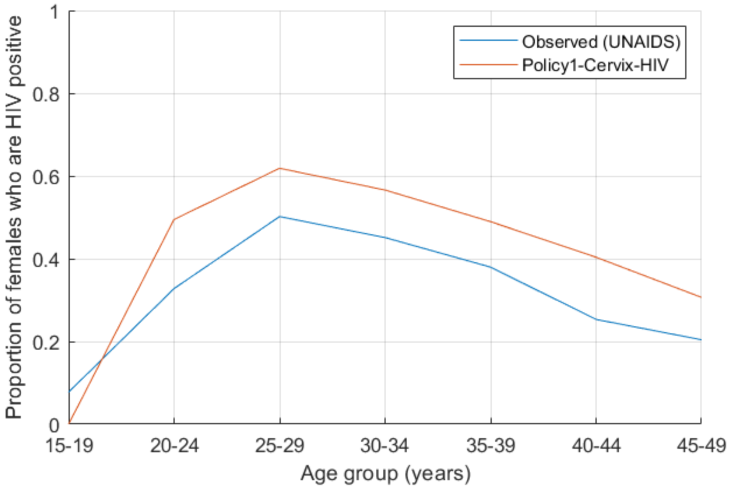

Figure S4: Observed versus simulated (Policy1-Cervix-HIV) prevalence of HIV in women, by age group, in 2005.

We report according to HPV-FRAME reporting standards for modelled evaluations of HPV prevention and control.[7]

I.c. Summary of key assumptions between models

Key differences between models included the number of age groups, number of high-risk (HR)-HPV group types modelled, assumptions on the magnitude of the association between HIV at different stages and HPV disease progression.

Table S1: Summary of key assumptions across models.

	DRIVE	***Policy1-Cervix-HIV***
**Setting**	• KwaZulu-Natal, South Africa	• KwaZulu-Natal, South Africa
**Age groups**	• 0-79 (16 x 5-year age groups)	• 5-79 (15 x 5-year age groups)
**HPV types**	• 2 groups HR types:<br>• HPV16/18/31/33/45/52/58<br>• Other HR HPV	• 3 groups HR types:<br>• HPV16/18<br>• HPV31/33/45/52/58<br>• Other HR HPV
**Natural immunity following HPV infection in women**	• 100% develop it (0-50% protection)<br>• Duration: 41 years	• 100% develop it (with 100% protection)<br>• Protection wanes over time (20% of protected individuals become susceptible per year)
**HIV/ HPV interactions**	• HIV increases susceptibility to HPV infection<br>• HIV reduces clearance of HPV<br>• HIV increases disease progression to cervical cancer<br>• HIV reduces disease regression from CIN2 or CIN3<br>• Magnitude of interactions are dependent of CD4 levels	• HIV increases susceptibility to HPV infection<br>• HIV reduces clearance of HPV<br>• HIV increases disease progression to cervical cancer<br>• HIV reduces disease regression from CIN1, CIN2, or CIN3<br>• HPV does not influence HIV susceptibility

	• HIV increases waning of HPV natural immunity (1.4-2.8 x shorter in PLHIV)<br>• HIV increases cervical cancer-associated mortality<br>• HPV does not influence HIV susceptibility	
**Condom coverage**	• Condoms introduced in 1995, scaled up linearly until 2000, then remains constant<br>• Condom use is uniform across risk groups<br>• The level of condom use from 2000 onwards (defined as the average proportion of the population that uses condoms times the percent of sexual acts for which those persons use a condom) is calibrated:<br>• Mean [Range] from the 25 best-fitting parameter sets = 28% [16-40%]	• Condom use is scaled up linearly from 0% in 1995 to to 23% by 2010, remaining constant thereafter
**VMMC coverage**	• VMMC at low, pre-HIV levels start at 1960 and have a 4% coverage among men aged 15-19, 6% coverage among men aged 20-24<br>• For VMMC scale-up scenarios, it is assumed that coverage increases linearly until 2000 and between 2000 and 2008 to match coverage levels estimated from SABSSM data[8,9]<br>• Following initiation of the national VMMC program in 2010, we model scale-up of circumcision for all men aged 15 or older at levels extrapolated backwards from 2012-2017 SABSSM and DHS data[8,9]	• VMMC at low, pre-HIV levels start at 1960 and have a coverage of 4%<br>• For VMMC scale-up scenarios, VMMC coverage increases from 4% to 48% of sexually active males across 2010 to 2020.
**ART coverage definition**	• ART coverage defines individuals who are on ART and virally supressed<br>• Assume no ART discontinuation<br>• Desired ART coverage can be set as an input to the model	• The model includes partial and full suppression with ART<br>• ART coverage defines the proportion of individuals who are fully virally suppressed<br>• Assume no ART discontinuation<br>• ART is an output to the model
**Effects of HIV interventions on HIV infection**	• Condoms reduce HIV acquisition in both males and females by 80%<br>• HIV-negative males with VMMC have 60% lower risk of acquiring HIV<br>• No effect of VMMC for men living with HIV, and no reduction in HIV transmission to female partners	• Condoms reduce HIV acquisition in both males and females by 90%<br>• HIV-negative males with VMMC have 60% lower risk of acquiring HIV<br>• No effect of VMMC for men living with HIV, and no reduction in HIV transmission to female partners
**Effect of ART on HPV/CC**	• ART reduces HPV infection risk of virally suppressed WLHIV to the same risk level as HIV negatives<br>• ART partially reduces HPV clearance, natural immunity waning, disease progression rates, disease regression rates, and cervical cancer-associated mortality of virally suppressed WLHIV to the same risk level as untreated WLHIV with >500 CD4 count	• ART reduces HPV infection risk of virally supressed WLHIV to near HIV-negative levels (95% return to base transition probability values)
**Effects of HIV interventions on HPV infection**	• Condoms reduce female HPV acquisition by 70% and male HPV acquisition by 46%<br>• Male circumcision does not affect HPV acquisition risk	• Condoms reduce female HPV acquisition by 70% and male HPV acquisition by 54%<br>• Male circumcision partially reduces HPV acquisition risk by 40%, accounting for decreased HPV persistence in circumcised men

**Cervical cancer and screening**	• Cervical cancer is classified as the number of true cervical cancer cases, regardless of whether they are detected<br>• Cervical cancer screening test performance is stratified for HIV-negative women, untreated women with HIV, and virally supressed women with HIV<br>• CIN treatment efficacy is stratified for HIV-negative women, untreated women with HIV, and virally supressed women with HIV	• Cervical cancer is classified as the number of cases detected by screening or symptoms<br>• Cervical screening test performance is stratified by underlying HPV health state, but not by HIV positivity or viral suppression status<br>• Precancer treatment efficacy is stratified for HIV-negative women, untreated women with HIV, and virally suppressed women with HIV

II. Model outcomes

II.a. Model fit to observed data

We compared cervical cancer incidence results between both models, as well as against the calibration target for cervical cancer incidence (GLOBOCAN 2018 estimates), as shown in Figure S5, for the status quo scenario in 2018. Both models produced similar results for crude cervical cancer incidence when stratified by HIV status. Policy1-Cervix-HIV does have minor declines in incidence for the 45-49 and 60-64 age groups as a result of screening applied at age groups applying an age-distribution for once-lifetime screening with cytology.

Both models were also compared against GLOBOCAN 2018 estimates. Calculated by the International Agency for Research on Cancer (IARC), GLOBOCAN cancer incidence estimates are based on available measures of cancer incidence, mortality, and mortality-to-incidence ratios.[10] GLOBOCAN estimates are available for South Africa, but are not available at the province-level. To approximate cervical cancer incidence in KZN rather than South Africa nationally, we adjusted the GLOBOCAN 2018 rates by age to take into account higher HIV prevalence in KZN. We assumed women with HIV have four times increased risk of cervical cancer[11] and then reweighted the overall cervical cancer incidence rate according to the HIV prevalence in KZN compared to SA nationally. HIV prevalence data in older ages are sparse, so we assumed prevalence is increased by the same proportion in women aged 50+. Overall, both models align well to the adjusted GLOBOCAN 2018 estimates for KZN.

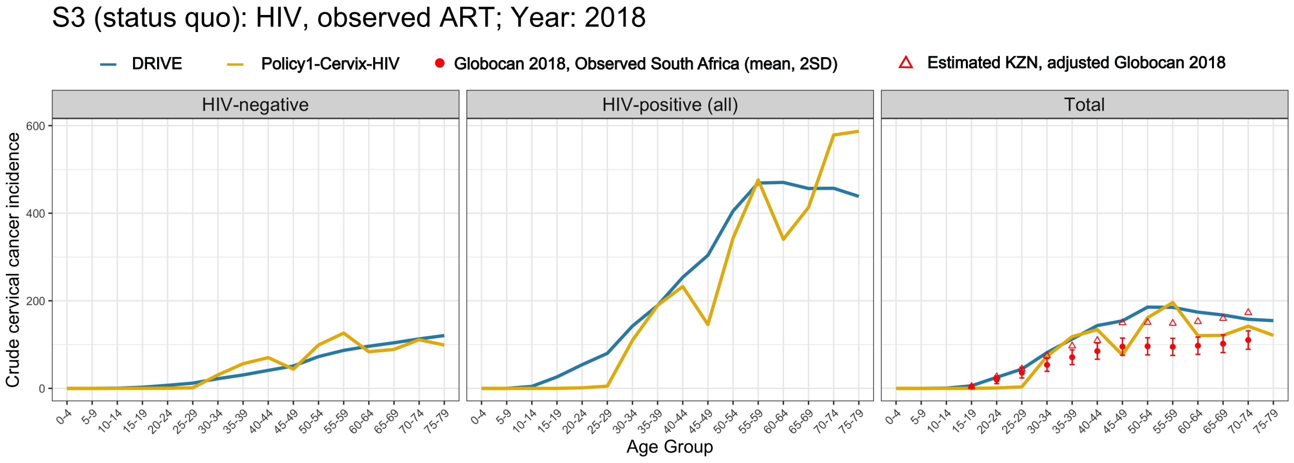

Figure S5:Crude cervical cancer incidence by age group for HIV-negative females, HIV-positive females, and all females combined in 2018 for the status quo scenario (observed HIV and cervical cancer interventions). Crude cervical cancer incidence for all females was fit to GLOBOCAN 2018 estimates for South Africa (solid circle) and the adjusted

estimate for KwaZulu-Natal, accounting for higher HPV prevalence (triangle). The GLOBOCAN estimates are shown as the mean, and error bars are shown as two standard deviations.

Both models were also fit to crude HPV prevalence estimates derived from McDonald, et al, as shown in Figure S6.[12] Note that both models did not calibrate to the HPV prevalence for age 15-19 because the observed data for this age range is an overestimate since McDonald, et al reports prevalence for age 17-19.[12]

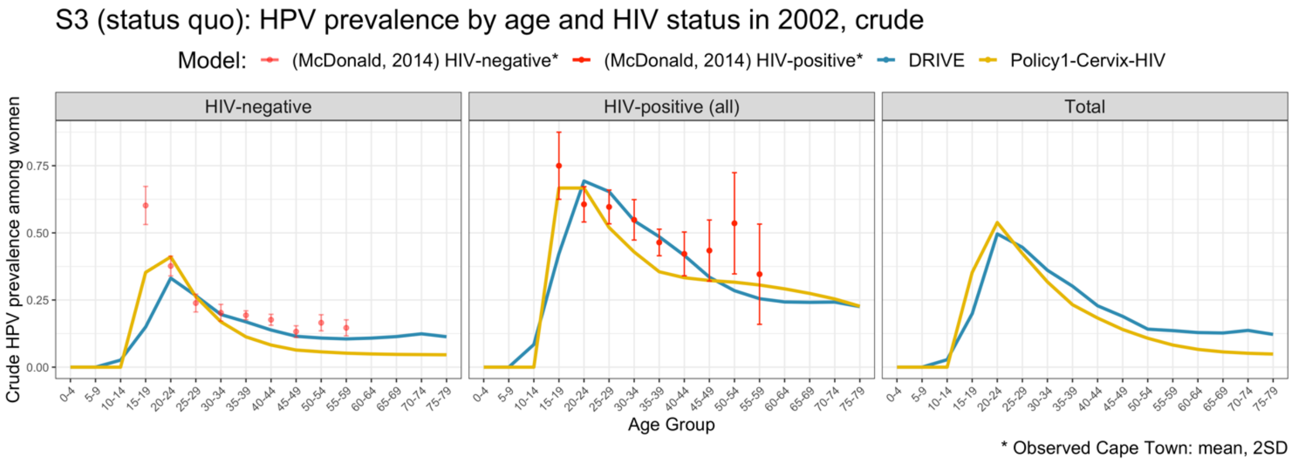

Figure S6: Model fit to observed HPV prevalence among women in 2002, stratified by HIV status and summarized for all women. Observed data is represented as a mean and error bars represent two standard deviations of the mean.

II.b. Model outcome comparisons

Figures S7 and S8 show the proportion of women who are on ART and virally suppressed from 2003 to 2124. Policy1-Cervix-HIV ART coverage is slightly lower than DRIVE due to differences in model structure. The DRIVE model can treat ART coverage as an input and an output, but the Policy1-Cervix-HIV model treats ART coverage as an output so for each cycle of the model, ART coverage is re-adjusted in the population to try and achieve the desired coverage level. Additionally, it is also important to note that Policy1-Cervix-HIV also models partial viral suppression; this incomplete viral suppression is assumed to reduce HIV mortality without conferring protection against HPV persistence or progression.

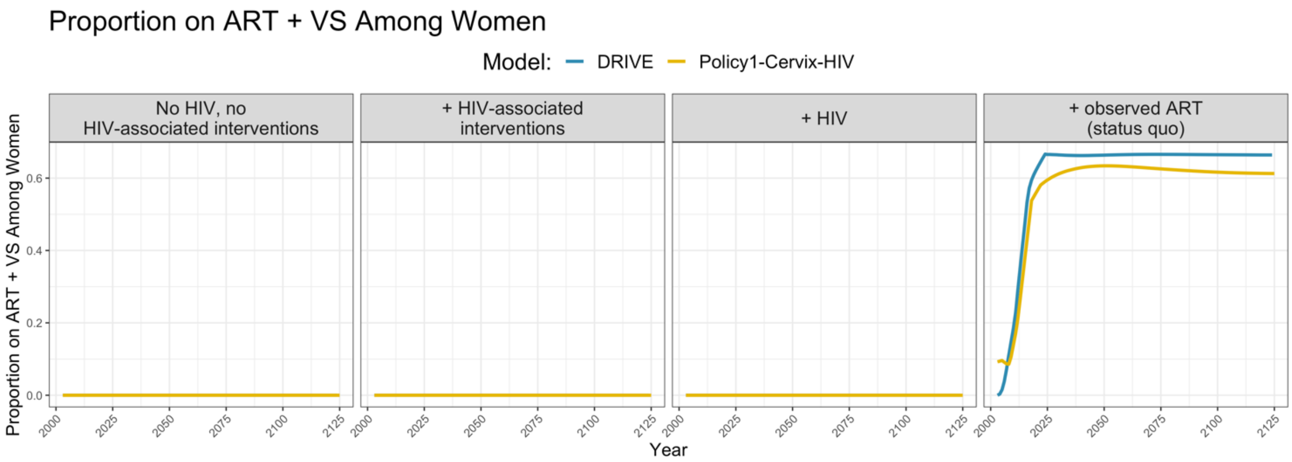

Figure S7: Proportion of women who are on ART and virally suppressed from 2003 to 2124 for the HIV-related intervention scenarios.

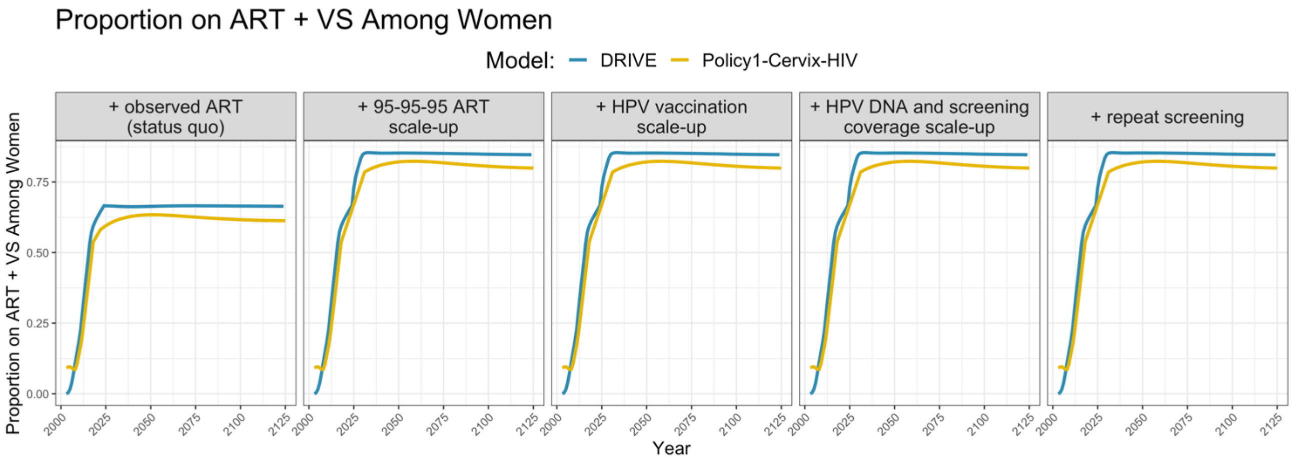

Figure S8: Proportion of women who are on ART and virally suppressed from 2003 to 2124 for the ART and cervical cancer intervention scale-up scenarios.

Figures S9 and S10 show the crude HIV prevalence from 2003 to 2124 for all scenarios. There are some differences between the two models that are worth noting. For the "+ HIV" scenario, HIV prevalence for DRIVE reaches a maximum around 2050, and then sees a decline in the subsequent years. Possible reasons for this may be the impact of that VMMC and condom coverage is having in reducing HIV prevalence over time, or the impact of infection dynamics in reducing the pool of individuals who are susceptible to HIV infection. It is worth noting that HIV prevalence does not decline lower than the HPV prevalence in 2024. Similarly, Policy1-Cervix-HIV sees a peak in HIV prevalence, but much earlier around 2005, suggesting that the impact of VMMC and condom coverage has a much faster impact in this model or that the infection dynamics reaches a peak in individual susceptible to HIV at an earlier time. For the remaining scenarios, both models trend similarly for crude HIV prevalence over time.

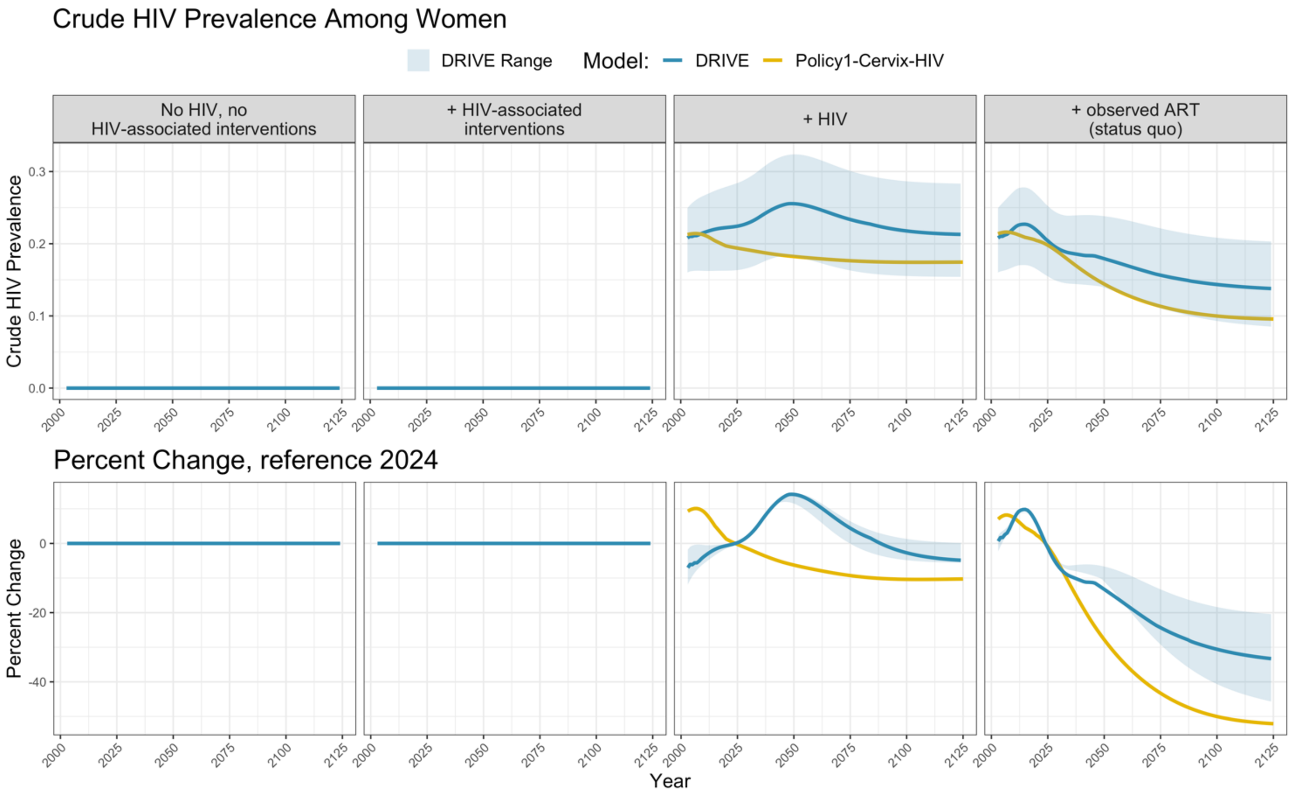

Figure S9: Crude HIV prevalence among women from 2003 to 2124 for the HIV-related intervention scenarios. The top panels show the crude HIV prevalence and the bottom panels show the percent change over time using the 2024 value as the reference. The shaded blue region represents the range of the 25 best-fitting parameter sets for the DRIVE model. The solid lines represent the median model results.

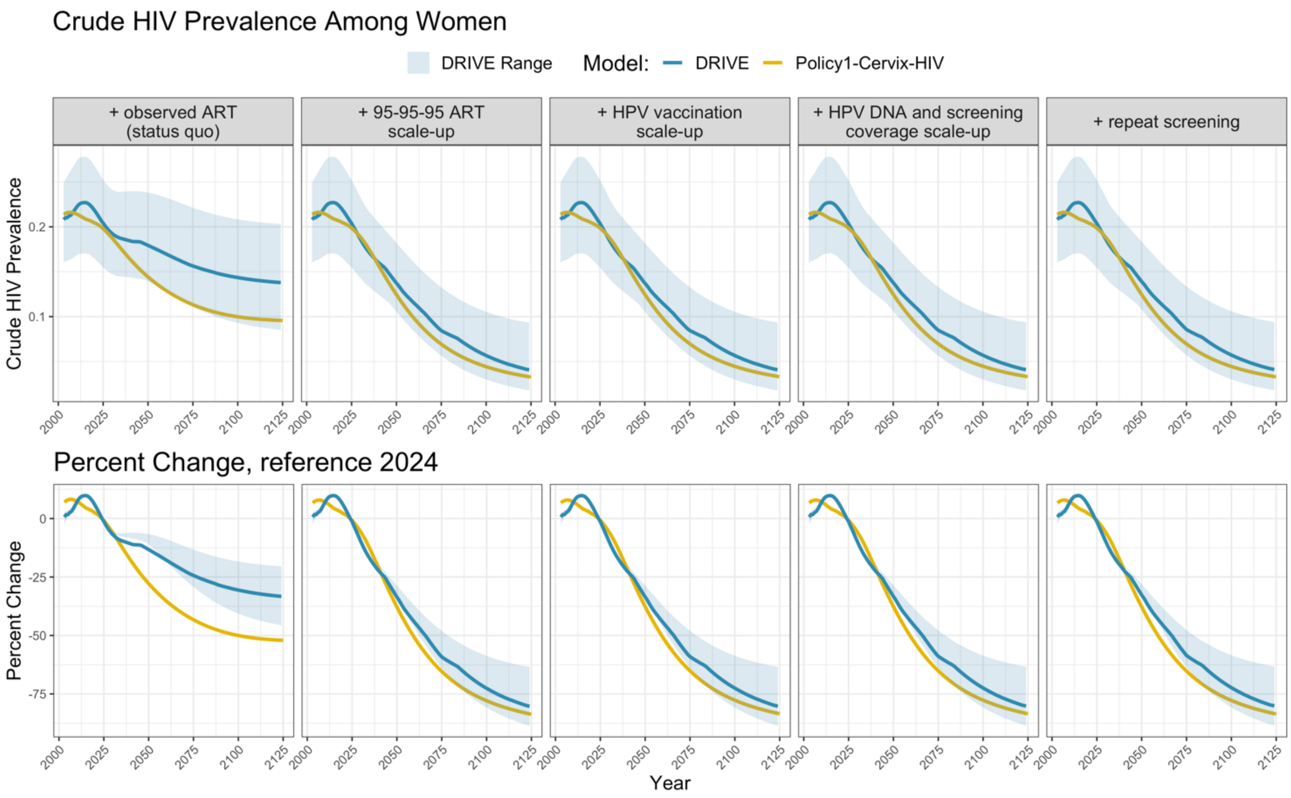

Figure S10: Crude HIV prevalence among women from 2003 to 2124 for the ART and cervical cancer intervention scale-up scenarios. The top panels show the crude HIV prevalence and the bottom panels show the percent change over time using the 2024 value as the reference. The shaded blue region represents the range of the 25 best-fitting parameter sets for the DRIVE model. The solid lines represent the median model results.

Figures S11 and S12 show the age-standardised HPV prevalence among women for all scenarios. For the HIV-related interventions scenarios (Figure S11), it is worth noting that HPV prevalence in Policy1-Cervix-HIV does not change as much in response to HIV-associated interventions, HIV, and ART compared to DRIVE. It remains relatively consistent across the four scenarios, with a slight decrease in the steady state HPV prevalence with the addition of HIV-associated interventions, a slight increase in steady state HPV prevalence with the addition of HIV, and a slight decrease in steady state HPV prevalence with the addition of ART. Differences between DRIVE and Policy1-Cervix-HIV predictions for age-standardised HPV prevalence in are likely due to differing assumptions regarding the efficacy of VMMC in protecting males against HPV acquisition.

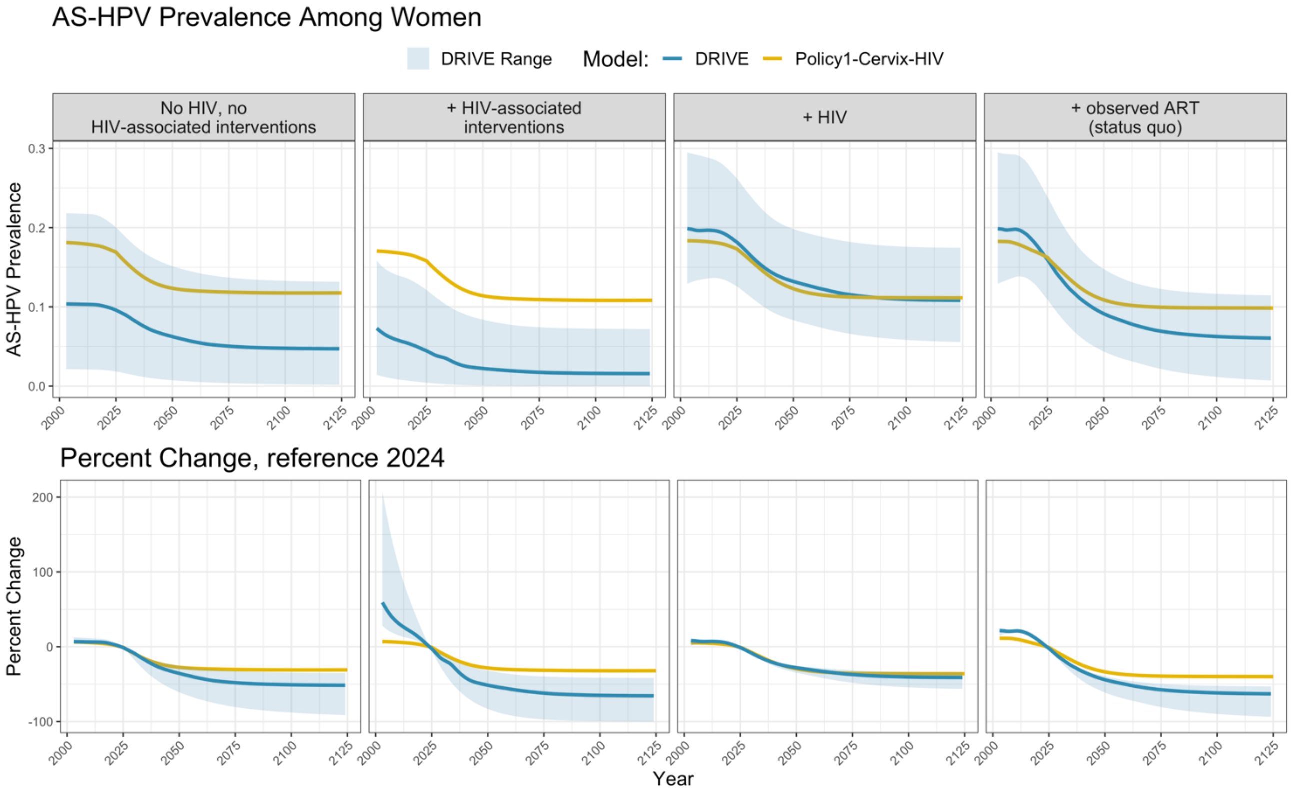

Figure S11: Age-standardised (AS) HPV prevalence among women from 2003 to 2124 for the HIV-related interventions scenarios. The top panels show the AS-HPV prevalence and the bottom panels show the percent change over time using the 2024 value as the reference. The shaded blue region represents the range of the 25 best-fitting parameter sets for the DRIVE model. The solid lines represent the median model results.

For the ART and cervical cancer intervention scale-up scenarios, each additional intervention is observed to lead to reductions in HPV prevalence in 2124. Overall, the HPV prevalence in Policy1-Cervix-HIV is greater than the median HPV prevalence observed in DRIVE.

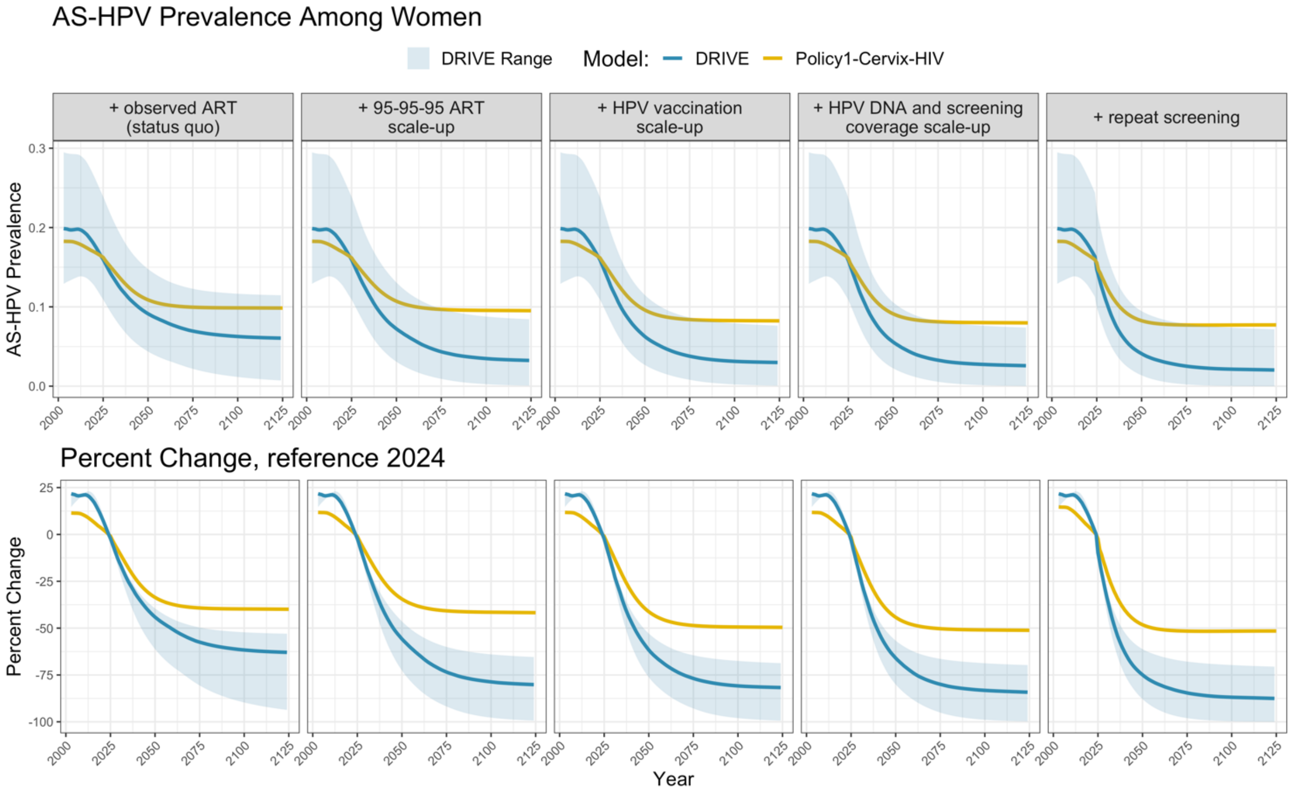

Figure S12: Age-standardised (AS) HPV prevalence among women from 2003 to 2124 for the ART and cervical cancer intervention scale-up scenarios. The top panels show the AS-HPV prevalence and the bottom panels show the percent change over time using the 2024 value as the reference. The shaded blue region represents the range of the 25 best-fitting parameter sets for the DRIVE model. The solid lines represent the median model results.

Figures S13 and S14 shows the age-standardised cervical cancer incidence over time for all scenarios. As described in the paper, the addition of ART in the "+ observed ART (status quo)" scenario shows how ART may temporarily increase the burden of cervical cancer, but incidence ultimately declines at a faster rate and reaches a lower point in 2124 than the no ART scenario ("+ HIV").

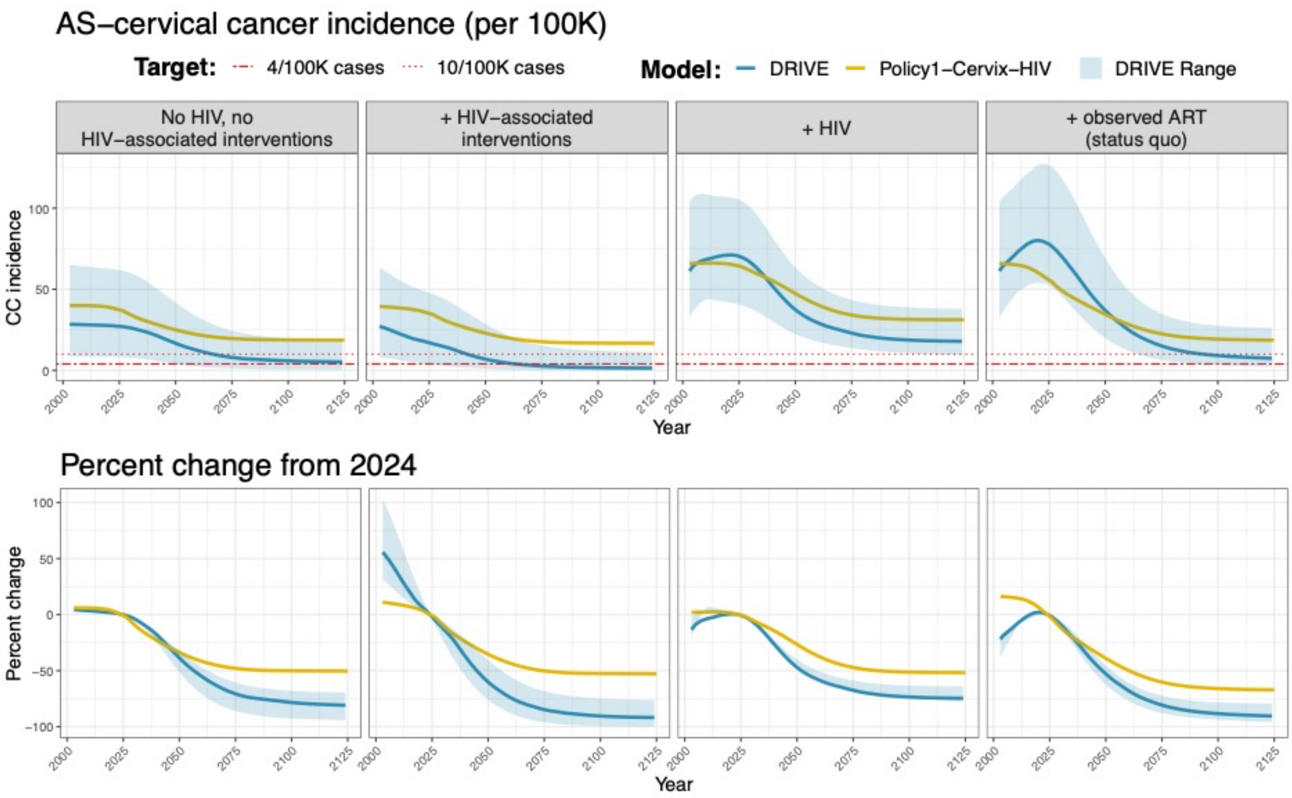

Figure S13: Age-standardised (AS) cervical cancer incidence from 2003 to 2124 for the HIV-related interventions scenarios. The top panels show the AS-cervical cancer incidence and the bottom panels show the percent change over time using the 2024 value as the reference. The shaded blue region represents the range of the 25 best-fitting parameter sets for the DRIVE model. The solid lines represent the median model results.

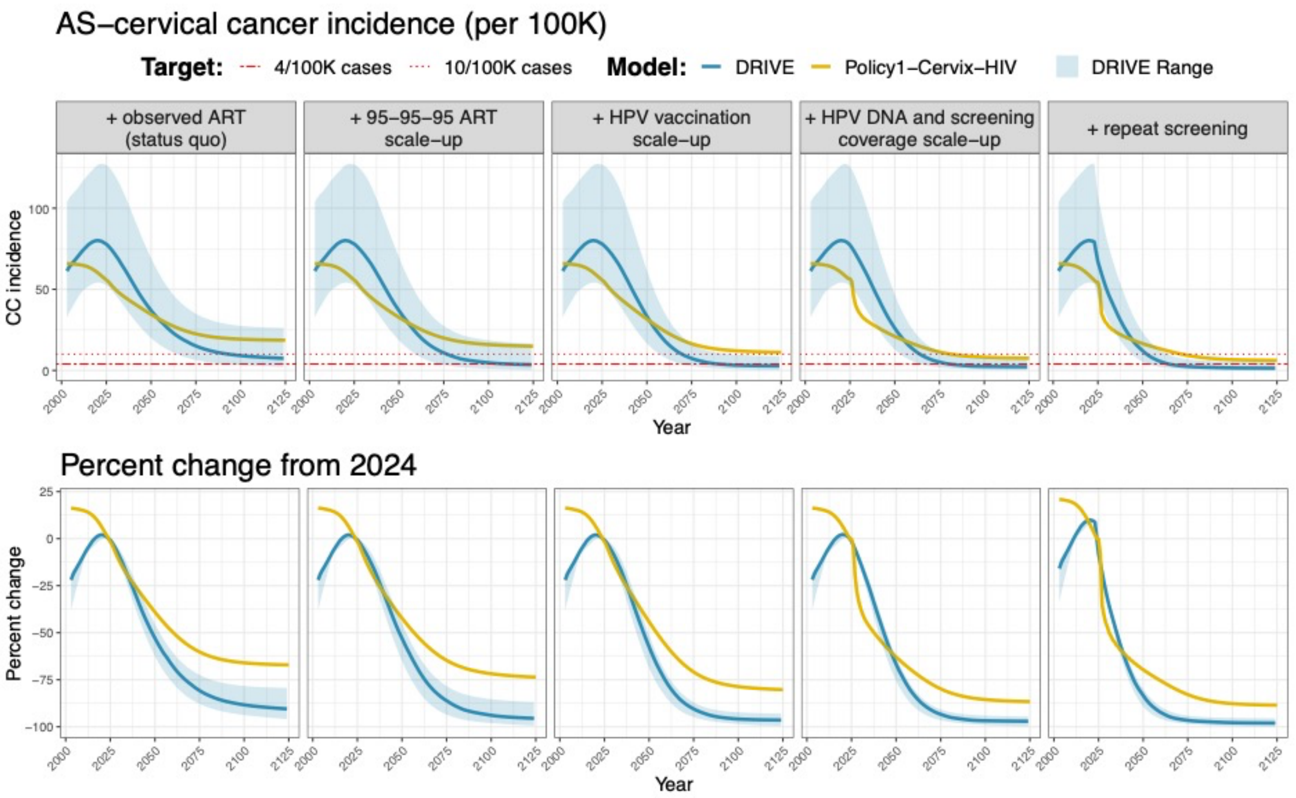

Figure S14: Age-standardised (AS) cervical cancer incidence from 2003 to 2124 for the ART and cervical cancer intervention scale-up scenarios. The top panels show the AS-cervical cancer incidence and the bottom panels show the percent change over time using the 2024 value as the reference. The shaded blue region represents the range of the 25 best-fitting parameter sets for the DRIVE model. The solid lines represent the median model results.

II.c. Sensitivity analyses

II.c.i. Policy1-Cervix-HIV Multivariate Sensitivity Analysis

To understand differences between DRIVE and Policy1-Cervix-HIV model predictions, and to assess the impact of key parameters on outcomes predicted by Policy1-Cervix-HIV a multivariate sensitivity analysis was conducted. Here, the impact of parameters governing cervical screening participation, distribution of vaccine-preventable HPV types, prevalence of VMMC and condom use, and the impact of HIV status on HPV natural history were assessed. Latin hypercube sampling was used to select 100 combinations of model parameters, with ranges as reported in Table S2.

Table S2. Parameter variations considered in multivariate sensitivity analysis for Policy1-Cervix-HIV.

Parameter	Lower bound	Upper bound
Cervical screening participation	2000-2003: scaled from 0% to 14%<br>2004-2016: scaled from 14% to 36%<br>2017- 2023: 36%<br>2023 onwards: remains at 36% for the status quo scenario, scaled to 53% over 2024-2030 for scenarios involving cervical screening scale-up	2000-2003: scaled from 0% to 23%<br>2004-2016: scaled from 23% to 60%<br>2017- 2023: 60%<br>2023 onwards: remains at 60% for the status quo scenario, scaled to 88% over 2024-2030 for scenarios involving cervical screening scale-up
Percent of possibly carcinogenic HPV infections preventable by HPV	77% (same assumption as baseline)	100% (extreme upper bound)

vaccination (based on prevalent HPV type-distribution prior to the introduction of HPV vaccination in KZN)		
Condom use (% of sexual partnerships consistently using condoms) and prevalence (%) of VMMC among sexually active males	Condom use from 1995 to 2010: scaled from 0% to 17%<br>Condom use from 2010 onwards: 17%<br>VMMC from 2010 to 2020: scaled from 0% to 36%<br>VMMC from 2021 onwards: 36%	Condom use from 1995 to 2010: scaled from 0% to 29%<br>Condom use from 2010 onwards: 29%<br>VMMC from 2010 to 2020: scaled from 0% to 60%<br>VMMC from 2021 onwards: 60%
Impact of HIV infection on HPV natural history	HIV assumed to have no impact on HPV natural history (extreme lower bound)	Same assumption as baseline where:<br>• HIV increases susceptibility to HPV infection<br>• HIV reduces clearance of HPV<br>• HIV increases disease progression to cervical cancer<br>• HIV reduces disease regression from CIN1, CIN2, or CIN3<br>• HPV does not influence HIV susceptibility

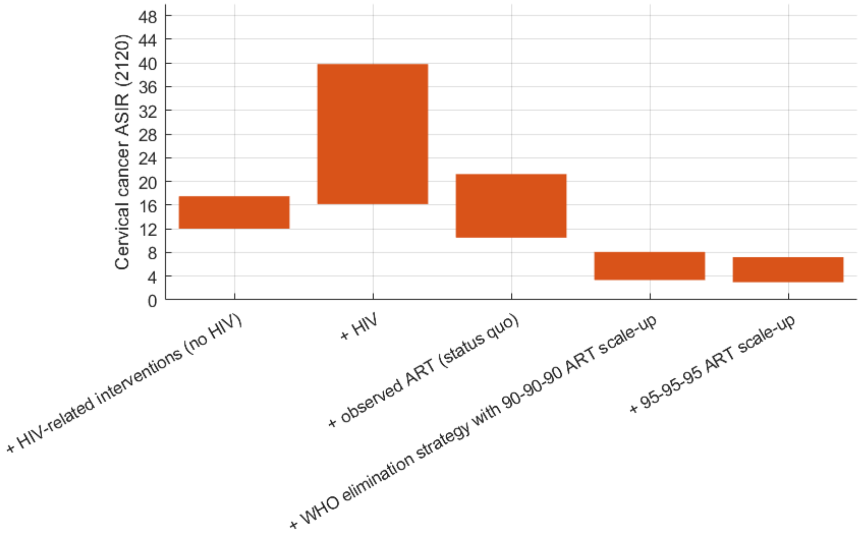

Figure S15: Age-standardized cervical cancer incidence rate in 2020 for key simulated scenarios, range generated by Latin Hypercube sampling of parameters described in Table S2.

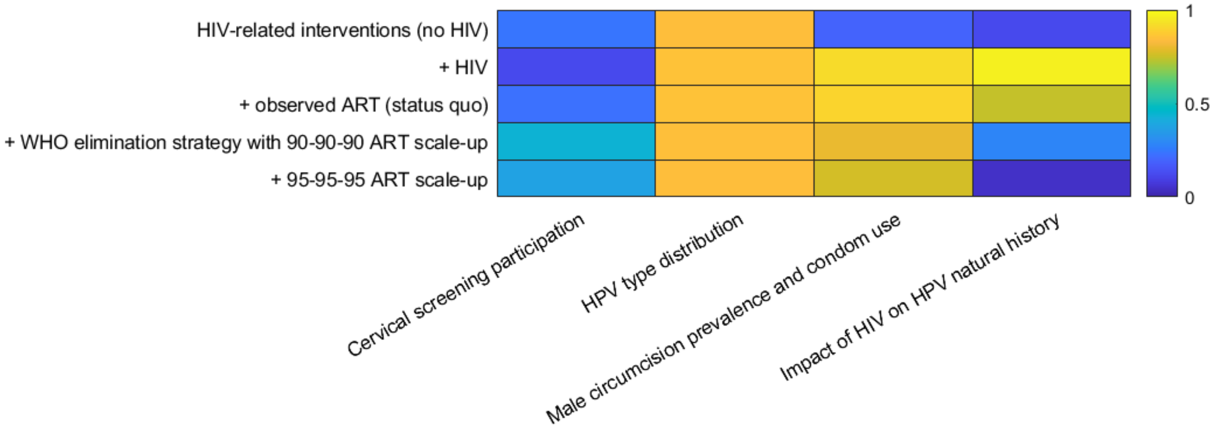

Figure S16: Correlation strength (partial rank correlation analysis) of cervical cancer ASIR in 2020 for key simulated scenarios against parameters varied in Latin Hypercube sampling.

III. Reporting

Our paper conforms to published guidance for reporting modeling studies of HPV prevention and economic evaluations – HPV-FRAME[7] and Consolidated Health Economic Evaluation Reporting Standards (CHEERS) 2022[13], respectively.

III.a. HPV-FRAME checklist: DRIVE Model

Inputs	Reported by age? (Y/N)	Reported by sex?	Comments
Target population for intervention	Y	Y	ART coverage for both males and females. HPV vaccination scale-up for girls age 9-14. Screening coverage scale up to twice lifetime for women without HIV and every five years for women living with HIV.
Sexual behavior	Y	Y	Sexual risk distribution reported by age in prior publication.[1]
Cohort examined for evaluation / time horizon	Y	Y	100-year time horizon from 2024-2124. Intervention is given to cohorts, but we examine the outcome in the population.
Quality of life assumptions	N	N	The article focused on epidemiological outcomes and did not focus on health economic outcomes.
Calibration	Y	Y	The model was calibrated with country-specific behavioral and epidemiological data (see prior publication).[1]
Validation (where possible)	Y	Y	The model was validated to HIV, HPV, and cervical cancer outcomes (see Appendix of prior publication).[1]

Costs	N	N	The article focused on epidemiological outcomes so it did not consider costs or health economic outcomes.
**Reporting standards for models of vaccination in adolescent individuals**			
Vaccine coverage	Y	Y	See Methods section of the article for information on the vaccine coverages modelled.
Vaccine efficacy	Y	Y	Assume 100% vaccine efficacy for two-dose HPV vaccination.
Vaccine cross-protection	N	N	We do not account for cross-protection against additional HPV types.
Duration vaccine protection and waning	N	N	We assume lifelong vaccine efficacy for the two-dose HPV vaccine.
Vaccine and delivery costs	N	N	The article focused on epidemiological outcomes so it did not consider costs or health economic outcomes.
Pre-vaccination disease burden	Y	Y	Pre-vaccination disease burden is reflected in the model validation, as shown in the appendix of a previous model publication.[1]
Duration of natural immunity	Y	Y	See appendix of a previous model publication.[1]
**Reporting standards for models of HPV prevention in LMIC**			
HIV prevalence rates, if endemic in country	Y	Y	The model was calibrated to HIV prevalence data stratified by age and sex (appendix of a previous model publication).[1]
Description of any opportunistic or pilot/demonstration screening projects ongoing	Y	Y	We ran scenarios reflecting current HIV prevention and treatment, and HPV/cervical cancer screening and vaccination strategies in South Africa (see section Methods of the article).
Costs	N	N	The article focused on epidemiological outcomes so it did not consider costs or health economic outcomes.
**Reporting standards for models of HPV-associated cancers among individuals living with HIV (ILWH)**			
HPV prevalence, CIN prevalence, and cervical cancer incidence by HIV status	Y	Y	Cervical cancer incidence is stratified by HIV status in the Results section of the article. HPV prevalence and cervical cancer incidence is also stratified by age in Section IIa of the Appendix.
HPV disease multipliers on HPV acquisition, progression from HPV infection to cancer for HIV-infected women/men	Y	Y	HPV acquisition multipliers for persons living with HIV is reported in the appendix of a previous publication. [1]
HPV-associated cancer mortality by HIV status	Y	Y	Cervical cancer-associated mortality rates by HIV health state

			are reported in the appendix of a previous publication. [1]
Relevant co-morbidities	Y	Y	Mortality rates account for HIV-associated mortality, cervical cancer-associated mortality, and background mortality due to other causes (see appendix of a previous publication).[1]
HPV-associated screening sensitivity/specificity by HIV status	N	Y	Screening test performance stratified by HIV status is reported in the appendix of a previous publication.[1] Test performance is assumed to be the same for all age groups.
**Reporting standards for models of cervical screening**			
Routine screening behavior	Y	Y	For the base-case scenario, we model once-per-lifetime cervical screening for women in the age range of 35-39 with cytology, colposcopy triage, and cryotherapy treatment (see Methods of the article). We also model scenarios of screening by HPV DNA, twice-lifetime screening for women without HIV, and every five years for women living with HIV.
Screening test(s) and colposcopy accuracies	N	Y	Accuracy of cryotherapy and colposcopy is reported in the appendix of a previous publication.[1] Accuracy is assumed to be the same for all age groups.
Abnormal test management	N	Y	Follow-up after an abnormal screening test is reported in the appendix of a previous publication.[1] Follow-up tests are not stratified by age.
Diagnostic follow-up of abnormal tests	N	Y	Follow-up after an abnormal screening test is reported in the appendix of a previous publication.[1] Follow-up tests are not stratified by age.
Management by disease grade	N	Y	Treatment is by cryotherapy for Scenarios 0 to 5. Scenario 6 and 7 involves treatment by thermal ablation and LLETZ. Management is consistent across all ages and disease grades. This is reported in the appendix of a previous publication.[1]
Sources of information for screening structure and parameterization	Y	Y	Screening structure in the model is described in the appendix of a previous publication.[1]
**Reporting standards for integrated models of cervical screening and vaccination**			
HPV type incidence, clearance, and progression rates	Y	Y	HPV natural history parameters stratified by age are reported in the

			appendix of a previous publication.[1]
Herd effect	Y	Y	The model is dynamic in natural history, so it captures population-level effects such as herd immunity.
Association between vaccination and screening uptake	Y	Y	The model assumes the same level of screening uptake by age regardless of vaccination status.
Screening test(s) and colposcopy accuracies	N	Y	Screening test performance and accuracy of colposcopy is reported in the appendix of a previous publication.[1] It is not stratified by age.
Fixed-variable costs	N	N	The article focused on epidemiological outcomes so it did not consider costs or health economic outcomes.
**B. Outputs**	**Reported by age? (Y/N)**	**Reported by sex?**	**Comments**
**Core reporting standards**			
Cancer incidence, mortality, life years, QALYs/DALYs (as appropriate)	Y	Y	Cancer incidence is reported in the Results section of the article. Cancer incidence is stratified by age for certain scenarios in Section IIa of the Appendix.
HPV prevalence, pre-intervention	N	Y	HPV outcomes pre-intervention are reported in Figure 1 of the Results section of the article.
CIN2/3 detected	N	Y	CIN outcomes were reported during model calibration, shown in the appendix of a previous publication.[1]
Sensitivity analysis on key inputs	N	Y	Results of sensitivity analyses are described in the appendix of a previous publication.[1]
Incremental cost-effectiveness ratios and costs saved	N	N	The article focused on epidemiological outcomes so it did not consider costs or health economic outcomes.
**Reporting standards for models of vaccination in adolescent individuals**			
Absolute reductions in HPV infections, and/or warts, post-vaccination	N	N	This was not presented since this study focuses on the impact of HIV and HPV interventions on the burden of cervical cancer.
Absolute reductions in CIN2+ post-vaccination	N	N	This was not presented since this study focuses on the impact of HIV and HPV interventions on the burden of cervical cancer.
Absolute reductions in invasive cancer (cervical and other HPV cancers, as relevant) post-vaccination	N	Y	We presented reduction in cervical incidence in Table 2 of the article. We do not present the results stratified by age.
**Reporting standards for models of HPV-associated cancers among individuals living with HIV (ILWH)**			

Reduction in cervical cancer incidence over time by HIV status (and CD4 count and ART status if modelled)	N	Y	Reduction in cervical cancer incidence over time by HIV status is presented in Figure 4 of the article.

III.b. HPV-FRAME checklist: Policy1-Cervix-HIV Model

Inputs	Reported by age? (Y/N)	Reported by sex?	Comments
Target population for intervention	Y	Y	ART coverage for both males and females. HPV vaccination scale-up for girls age 9-14. Screening coverage scale up to twice lifetime for women without HIV and every five years for women living with HIV.
Sexual behavior	Y	Y	Reported in appendix section I.
Cohort examined for evaluation / time horizon	Y	Y	100-year time horizon from 2024-2124. Intervention is given to cohorts, but we examine the outcome in the population.
Quality of life assumptions	N	N	The article focused on epidemiological outcomes and did not focus on health economic outcomes.
Calibration	Y	Y	The model was calibrated with country-specific epidemiological data (see fit to HPV prevalence in section IIa)
Validation (where possible)	Y	Y	The model was validated with country-specific epidemiological data (see fit to cervical cancer incidence in section IIa)
Costs	N	N	The article focused on epidemiological outcomes so it did not consider costs or health economic outcomes.
**Reporting standards for models of vaccination in adolescent individuals**			
Vaccine coverage	Y	Y	See Methods section of the article for information on the vaccine coverages modelled.
Vaccine efficacy	Y	Y	Assume 100% vaccine efficacy for two-dose HPV vaccination.
Vaccine cross-protection	N	N	We do not account for cross-protection against additional HPV types.
Duration vaccine protection and waning	N	N	We assume lifelong vaccine efficacy for the two-dose HPV vaccine.
Vaccine and delivery costs	N	N	The article focused on epidemiological outcomes so it did not consider costs or health economic outcomes.
Pre-vaccination disease burden	Y	Y	Pre-vaccination disease burden is reflected in the model validation, as

			shown in the appendix of a previous model publication.[1]
Duration of natural immunity	Y	Y	See appendix section I.
**Reporting standards for models of HPV prevention in LMIC**			
HIV prevalence rates, if endemic in country	Y	Y	The model was calibrated to annual HIV prevalence in females aged 15-49 years as reported in appendix section I.
Description of any opportunistic or pilot/demonstration screening projects ongoing	Y	Y	We ran scenarios reflecting current HIV prevention and treatment, and HPV/cervical cancer screening and vaccination strategies in South Africa (see section Methods of the article).
Costs	N	N	The article focused on epidemiological outcomes so it did not consider costs or health economic outcomes.
**Reporting standards for models of HPV-associated cancers among individuals living with HIV (ILWH)**			
HPV prevalence, CIN prevalence, and cervical cancer incidence by HIV status	Y	Y	Cervical cancer incidence is stratified by HIV status in the Results section of the article. HPV prevalence and cervical cancer incidence is also stratified by age in Section II of the Appendix.
HPV disease multipliers on HPV acquisition, progression from HPV infection to cancer for HIV-infected women/men	Y	Y	HPV acquisition multipliers for persons living with HIV is reported in the appendix of a previous publication.[6]
HPV-associated cancer mortality by HIV status	Y	Y	Cervical cancer mortality for women living with HIV is assumed to be the same as women in the general population.
Relevant co-morbidities	N	N	Additional co-morbidities are not considered.
HPV-associated screening sensitivity/specificity by HIV status	N	Y	Screening test performance is assumed to be the same for women living with HIV and women in the general population. Screening test performance is described in a prior publication.[5]
**Reporting standards for models of cervical screening**			
Routine screening behavior	Y	Y	For the base-case scenario, we model once-per-lifetime cervical screening for women in the age range of 35-39 with cytology, colposcopy triage, and cryotherapy treatment (see Methods of the article). We also model scenarios of screening by HPV DNA, twice-lifetime screening for women without HIV, and every five years for women living with HIV.

Screening test(s) and colposcopy accuracies	N	Y	Accuracy of cryotherapy and colposcopy is reported in a previous publication.[5]
Abnormal test management	N	Y	Follow-up after an abnormal screening test is reported in the appendix of a previous publication.[1] Follow-up tests are not stratified by age.
Diagnostic follow-up of abnormal tests	N	Y	Follow-up after an abnormal screening test is reported in the appendix of a previous publication.[1] Follow-up tests are not stratified by age.
Management by disease grade	N	Y	Treatment is by cryotherapy for Scenarios 0 to 5. Scenario 6 and 7 involves treatment by thermal ablation and LLETZ. Management is consistent across all ages and disease grades. This is reported in the appendix of a previous publication.[1]
Sources of information for screening structure and parameterization	Y	Y	Screening structure in the model is described in the appendix of a previous publication.[1]
**Reporting standards for integrated models of cervical screening and vaccination**			
HPV type incidence, clearance, and progression rates	Y	Y	HPV natural history parameters stratified by age are reported in the appendix of a previous publication.[6]
Herd effect	Y	Y	The model is dynamic in natural history, so it captures population-level effects such as herd immunity.
Association between vaccination and screening uptake	Y	Y	The model assumes the same level of screening uptake by age regardless of vaccination status.
Screening test(s) and colposcopy accuracies	N	Y	Screening test performance and accuracy of colposcopy is reported in the appendix of a previous publication.[5]
Fixed-variable costs	N	N	The article focused on epidemiological outcomes so it did not consider costs or health economic outcomes.
**B. Outputs**	**Reported by age? (Y/N)**	**Reported by sex?**	**Comments**
**Core reporting standards**			
Cancer incidence, mortality, life years, QALYs/DALYs (as appropriate)	Y	Y	Cancer incidence is reported in the Results section of the article. Cancer incidence is stratified by age for certain scenarios in Section IIa of the Appendix.
HPV prevalence, pre-intervention	N	Y	HPV outcomes pre-intervention are reported in Figure 1 of the Results section of the article.

CIN2/3 detected	N	N	CIN outcomes were not reported.
Sensitivity analysis on key inputs	N	Y	Results of sensitivity analyses are reported in appendix section IIc.
Incremental cost-effectiveness ratios and costs saved	N	N	The article focused on epidemiological outcomes so it did not consider costs or health economic outcomes.
**Reporting standards for models of vaccination in adolescent individuals**			
Absolute reductions in HPV infections, and/or warts, post-vaccination	N	N	This was not presented since this study focuses on the impact of HIV and HPV interventions on the burden of cervical cancer.
Absolute reductions in CIN2+ post-vaccination	N	N	This was not presented since this study focuses on the impact of HIV and HPV interventions on the burden of cervical cancer.
Absolute reductions in invasive cancer (cervical and other HPV cancers, as relevant) post-vaccination	N	Y	We presented reduction in cervical incidence in Table 2 of the article. We do not present the results stratified by age.
**Reporting standards for models of HPV-associated cancers among individuals living with HIV (ILWH)**			
Reduction in cervical cancer incidence over time by HIV status (and CD4 count and ART status if modelled)	N	Y	Reduction in cervical cancer incidence over time by HIV status is presented in Figure 4 of the article.

VI. References

1. Tran J, Hathaway CL, Broshkevitch CJ, et al. Cost-effectiveness of single-visit cervical cancer screening in KwaZulu-Natal, South Africa: a model-based analysis accounting for the HIV epidemic. *Front Oncol* 2024; **14**: 1382599.

2. Hall MT, Smith MA, Simms KT, Barnabas RV, Canfell K, Murray JM. The past, present and future impact of HIV prevention and control on HPV and cervical disease in Tanzania: a modelling study. *PLoS One* 2020; **15(5): e0231388**.

3. Hall MT, Smith MA, Simms KT, Barnabas R, Murray JM, Canfell K. Elimination of cervical cancer in Tanzania: Modelled analysis of elimination in the context of endemic HIV infection and active HIV control. *International journal of cancer* 2021; **149**(2): 297-306.

4. Hall MT, Simms KT, Murray JM, et al. Benefits and harms of cervical screening, triage and treatment strategies in women living with HIV. *Nature Medicine* 2023; **29**(12): 3059-66.

5. Hall MT, Smith MA, Simms KT, Barnabas R, Murray JM, Canfell K. Elimination of cervical cancer in Tanzania: Modelled analysis of elimination in the context of endemic HIV infection and active HIV control. *Int J Cancer* 2021; **149**(2): 297-306.

6. Hall MT, Smith MA, Simms KT, Barnabas RV, Canfell K, Murray JM. The past, present and future impact of HIV prevention and control on HPV and cervical disease in Tanzania: A modelling study. *PLoS One* 2020; **15**(5): e0231388.

7. Canfell K, Kim JJ, Kulasingam S, et al. HPV-FRAME: A consensus statement and quality framework for modelled evaluations of HPV-related cancer control. *Papillomavirus Res* 2019; **8**: 100184.

8. Shisana O SL RT, Zungu NP, Zuma K, Ngogo N, Jooste S, PillayVan Wyk V, Parker W, Pezi S, Davids A, Nwanyanwu O, Dinh TH, SABSSM III Implementation Team. South African National HIV Prevalence, Incidence, Behaviour and Communication Survey, 2008: The health of our children. Cape Town, 2010.

9. Connolly C, Simbayi LC, Shanmugam R, Nqeketo A. Male circumcision and its relationship to HIV infection in South Africa: results of a national survey in 2002. *S Afr Med J* 2008; **98**(10): 789-94.
10. Bray F, Ferlay J, Soerjomataram I, Siegel RL, Torre LA, Jemal A. Global cancer statistics 2018: GLOBOCAN estimates of incidence and mortality worldwide for 36 cancers in 185 countries. *CA Cancer J Clin* 2018; **68**(6): 394-424.
11. Kong X, Wang MC, Gray R. Analysis of longitudinal multivariate outcome data from couples cohort studies: application to HPV transmission dynamics. *J Am Stat Assoc* 2015; **110**(510): 472-85.
12. McDonald AC, Tergas AI, Kuhn L, Denny L, Wright TC, Jr. Distribution of Human Papillomavirus Genotypes among HIV-Positive and HIV-Negative Women in Cape Town, South Africa. *Front Oncol* 2014; **4**: 48.
13. Husereau D, Drummond M, Augustovski F, et al. Consolidated Health Economic Evaluation Reporting Standards 2022 (CHEERS 2022) Statement: Updated Reporting Guidance for Health Economic Evaluations. *Value in Health* 2022; **25**(1): 3-9.
